# CHASE-independent cytokinin perception triggers 3′,5′-cAMP signaling in *Sinorhizobium meliloti*

**DOI:** 10.1128/jb.00585-25

**Published:** 2026-02-25

**Authors:** Niklas M. Schäfer, Elizaveta Krol, Nicole Paczia, Neda Farmani, Anke Becker

**Affiliations:** 1 Department of Biology and Center for Synthetic Microbiology (SYNMIKRO), Philipps-Universität Marburg 9377, Marburg, Germany; 2 Max Planck Institute for Terrestrial Microbiology 28310, Marburg, Germany; Dartmouth College Geisel School of Medicine, Hanover, New Hampshire, USA

**Keywords:** second messenger, cyclic nucleotides, adenosine 3',5'-cyclic monophosphate, adenylate cyclase, 1-deoxyxylulose-5-phosphate synthase, cytokinins, isopentenyladenine, *Sinorhizobium meliloti*

## Abstract

The *Medicago sativa-Sinorhizobium meliloti* symbiotic plant-microbe interaction, which results in the formation of nitrogen-fixing root nodules, is subject to sophisticated genetic and metabolic regulation by both partners. *S. meliloti* is capable of inhibiting secondary plant infections via an adenosine 3′,5′-cyclic adenosine monophosphate (cAMP)-dependent regulatory pathway that depends on CHASE2 domain adenylate/guanylate cyclases (AC/GCs). This pathway likely responds to a plant signal of protein nature. Plant cytokinins (CKs) are adenine derivative phytohormones that control many aspects of plant development, including the symbiotic nodule formation. Classical CK receptors in plants and bacteria contain a CHASE domain. In our study, we present a novel, CK-dependent cAMP signaling pathway, specifically mediated by the AC/GC CyaB, which lacks any known receptor domains. The plant CKs N^6^(Δ^2^isopentenyl)-adenine (iP), trans-zeatin, kinetin, and 6-benzylaminopurine all promoted CyaB-dependent increase in cAMP levels detected through a genetic reporter construct. Among these four CKs, iP exerted the strongest effect. Metabolic profiling confirmed the CyaB-dependent accumulation of cAMP in *S. meliloti* cells, cultured in the presence of iP. The first enzyme in the terpenoid biosynthetic pathway, 1-deoxyxylulose-5-phosphate synthase Dxs, was identified as a CyaB interaction partner and is proposed to mediate the CK perception. CyaB homologs from closely related members of the *Rhizobiaceae* were able to interact with Dxs and to mediate cAMP signaling in response to iP.

**IMPORTANCE:**

Symbiotic interactions between nitrogen-fixing bacteria and leguminous plants are important for agriculture, ecological sustainability, and human nutrition. Maintaining an optimal number of symbiotic infections per plant is crucial for efficient symbiosis. Previous studies have shown that *S. meliloti* 3′,5′-cyclic adenosine monophosphate (cAMP) signaling mediates the inhibition of secondary symbiotic infections of *Medicago* plants. We discovered a molecular mechanism that allows the symbiotic bacterium *Sinorhizobium meliloti* to respond to the *Medicago* plant adenosine derivative phytohormones named cytokinins (CKs) via cAMP signaling. This mechanism is mediated by the adenylate/guanylate cyclase CyaB. CyaB lacks any sensory domains and may perceive the CKs via its interaction partner deoxyxylulose-5-phosphate synthase Dxs.

## INTRODUCTION

Cyclic nucleotide 3′5′-cyclic adenosine monophosphate (cAMP) is one of the most ubiquitous second messengers in prokaryotes and eukaryotes. It is involved in a wide variety of functions including carbon and amino acid metabolism, surface structures, and virulence (reviewed in reference 1). In addition, there is increasing evidence for a role of cAMP in bacterial antimicrobial resistance (reviewed in reference 2). cAMP exerts its effects primarily by activating DNA-binding proteins of the CRP family, which regulate gene transcription (reviewed in reference 3).

The biosynthesis of cAMP from ATP is mediated by adenylate/guanylate cyclases (AC/GCs). The number of such enzymes varies in different bacterial species. The soil-dwelling symbiotic bacterium *Sinorhizobium meliloti* possesses 28 annotated type III AC/GCs, suggesting a high versatility of cAMP functions in this bacterium (4). In symbiosis with its host plants of the genus *Medicago*, *S. meliloti* greatly enhances plant nitrogen nutrition through nitrogen fixation in root nodules. Symbiotic nitrogen fixation is a complex, highly energy-demanding process. Therefore, the number of successful plant infections leading to root nodule formation is tightly controlled by the plant through a systemic mechanism, designated autoinhibition of nodulation (AON) (5). *S. meliloti* additionally regulates the number of symbiotic events via a cAMP-dependent mechanism, contributing to restriction of secondary infections on plants that already initiated the symbiotic nodule development (6). At the early stages of nodule formation, bacteria infect the root hair cells, propagate within an infection thread, and ultimately invade the root cortex cells in the nodule primordium (reviewed in reference 7, 8). The number of the secondary root hair infection events is controlled by the *S. meliloti c*yclase/*h*istidine kinase*-a*ssociated *s*ensory *e*xtracellular (CHASE2) domain AC/GCs CyaD1, CyaD2, and CyaK and the CRP family transcription regulator Clr (6). The cAMP biosynthesis by CHASE2 domain AC/GCs increases upon sensing of the plant ribosomal protein uL2 through the outer membrane β-barrel protein NsrA (9, 10). Elevated cAMP activates the Clr regulon, which is required to prevent excessive symbiotic infections (9, 11, 12).

On the plant side, AON is governed by a set of signaling molecules including N^6^-adenine derivative cytokinins (CKs). The adenine base of CKs carries an isoprenoid, aromatic, or furfural side chain and is either free or conjugated to a sugar. The actions of these phytohormones are opposite to those of auxins and mediate regulation of plant environmental responses, immunity, growth, and development, including the formation of symbiotic root nodules (reviewed in reference 13, 14). Isopentenyladenine (iP) and its hydroxylated derivative trans-zeatin (tZ) are common plant CKs with an isoprenoid side chain (15). Although very similar in their structure, tZ and iP are synthesized in plant roots and shoots, respectively, and are used for communication between these two plant organs (16). Aromatic and furfural CKs like 6-benzylaminopurine (6-BAP) and kinetin also show biological activity in plants and were used in studies of plant-microbe interactions (17, 18). Local activation of the plant root CK biosynthesis in response to the bacterial symbiotic signals and activation of root nodule development upon exogenous application of CKs are well documented (17, 19–23). Moreover, CK treatment has been shown to increase the expression of a *Medicago truncatula* gene encoding a regulatory peptide, which is then transported via the xylem to the shoots (24). The homologous *Lotus japonicus* regulatory peptide and its shoot receptor activate production of the shoot CKs, which are transported back to the roots via the phloem and mediate the AON (25, 26).

Considering their role in plant-microbe interactions, it is not surprising that CKs can also be produced or sensed by plant-associated bacteria. The plant pathogen *Agrobacterium fabrum* (formerly *Agrobacterium tumefaciens*) uses transfer DNA to introduce genes for CK and auxin biosynthesis into the plant cells, thereby manipulating the phytohormone household of the infected tissue and promoting crown gall tumor formation (27). The ability to synthesize CKs has been demonstrated in plant-pathogenic species of *Pseudomonas*, *Xanthomonas*, and *Rhodococcus* (15, 28). The first bacterial CK receptor, the CHASE domain histidine kinase PcrK, was identified in the black rot pathogen *Xanthomonas campestris*. PcrK perceives CKs and responds with autophosphorylation, promoting a physiological response that includes increased oxidative stress tolerance and virulence (29, 30). In laboratory conditions, legume symbionts including *S. meliloti* are able to produce CKs in the range of tens to hundreds of molecules per bacterial cell (31, 32).

In this work, we analyze the ability of *S. meliloti* to sense plant CK phytohormones and establish a regulatory connection to the cAMP-mediated AON-relevant bacterial traits. Using genetic methods and co-immunoprecipitation (CoIP) assays, we identify the AC CyaB and its interaction partner Dxs as potential CK receptors. Furthermore, we provide evidence that this CK perception mechanism is conserved in related plant-interacting *Rhizobiaceae* bacteria.

## RESULTS

### Plant CKs trigger cAMP biosynthesis by CyaB

**Fig 1:**
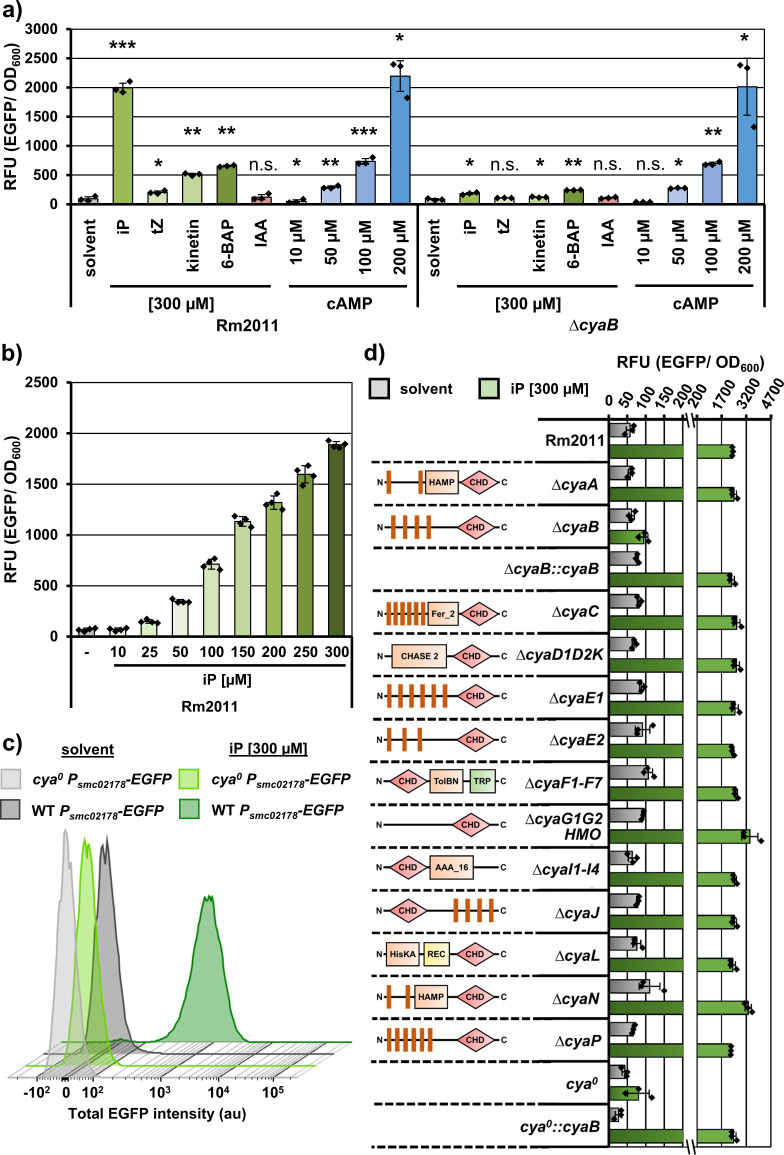
*S. meliloti* Rm2011 responds to CKs with CyaB-mediated activation of the fluorescent cAMP reporter *smc02178-EGFP*. EGFP reporter fluorescence of *S. meliloti* strains, carrying the reporter plasmid pSRKKm-smc02178-EGFP. The strains were grown for 24 h in Vincent minimal medium supplemented with the indicated substances. The error bars represent the standard deviation of three to four biological replicates, and the diamonds show the single values. (**a**) Reporter response to phytohormones and cAMP in the wild-type strain Rm2011 and its △*cyaB* derivative. Asterisks indicate significance of the differences determined in Student’s *t*-test. (***): *P* < 0.0005, (**): *P* < 0.005, (*): *P* < 0.05, n.s.: not significant. (**b**) Reporter response in the wild-type strain Rm2011 to increasing concentrations of iP. (**c**) Flow-cytometry analysis (FACS) of the Rm2011 wild type and the *cya^0^* strain. Histograms show the EGFP signal of one representative of the three analyzed biological replicates. For each replicate, 28,000 events or more were detected. (**d**) Identification of CyaB as an AC/GC responsible for reporter response to CK phytohormones. The graph shows reporter response to 300 µM iP in the wild-type strain Rm2011, strains with deletions in single or multiple AC/GC genes, and strains △*cyaB* and *cya^0^*, complemented with *cyaB*. The strain △*cyaD1D2K* is lacking CyaD1, CyaD2, and CyaK; the strain △*cyaF1-F7* is lacking CyaF1, CyaF2, CyaF3, CyaF4, CyaF5, CyaF6, and CyaF7; the strain △*cyaG1G2HMO* is lacking CyaG1, CyaG2, CyaH, CyaM, and CyaO; the strain △*cyaI1-I4* is lacking CyaI1, CyaI2, CyaI3, and CyaI4.

To evaluate the effect of commercially available plant CKs on cAMP synthesis in *S. meliloti* Rm2011, we employed the reporter plasmid pSRKKm-smc02178-EGFP, which was previously used to detect cAMP in *S. meliloti* (11). This reporter is reliably activated dependent on Clr and endogenous or exogenous cAMP, resulting in EGFP fluorescence (6, 11). We tested the response to exogenously applied iP, tZ, kinetin, and 6-BAP, using cAMP as a positive control. Since auxins play a role as CK antagonists in plant development (33), we also tested the auxin phytohormone indole-3-acetic acid (IAA). The cultures, supplemented with the test substances, were grown in Vincent minimal medium and reached an early stationary growth phase at the time point of fluorescence measurement. Addition of tZ, kinetin, 6-BAP, or iP resulted in increased EGFP fluorescence, whereas no response to IAA was detected (Fig. 1a). Since iP exerted the strongest effect, comparable to that of exogenously supplemented 200 µM cAMP, we selected this CK for further investigation. The response to iP was dose-dependent, with 25 µM defined as the lowest concentration capable of promoting reliable reporter activation when added to the cultures (Fig. 1b). Flow cytometry analysis revealed that 98% of the iP-treated wild-type cells carrying pSRKKm-smc02178-EGFP belonged to a distinct high fluorescence population, indicating a reliable, homogeneous Clr-cAMP-dependent promoter activation in the cell population (Fig. 1c). Moreover, the strain *cya*^0^, lacking all the 28 class III AC/GC genes annotated in the *S. meliloti* Rm2011 genome, failed to respond to iP with an increase in EGFP fluorescence, suggesting that the iP signaling is mediated by at least one of these encoded AC/GC enzymes (Fig. 1c and d).

To identify the AC/GC, responsible for the observed effect, we screened a collection of mutants, containing strains with single or multiple deletions in AC/GC genes, for their ability to respond to iP with activation of the *smc02178-EGFP* reporter. Out of 13 strains, carrying AC/GC gene deletions, only the *∆cyaB* strain showed a strongly reduced iP response although the reporter was readily activated when cAMP was added (Fig. 1a and d). This suggested that cAMP biosynthesis by CyaB is activated in the presence of iP. The *∆cyaB* strain also lost the ability to activate *smc02178-EGFP* expression when grown with tZ, kinetin, or 6-BAP (Fig. 1a), implying that CyaB may mediate the sensing of various plant CKs. Reintegration of the *cyaB* gene at its native chromosomal locus in either the *∆cyaB* or *cya^0^* strain restored the cAMP-dependent iP response comparable to that of the Rm2011 wild type (Fig. 1d).

**Fig 2:**
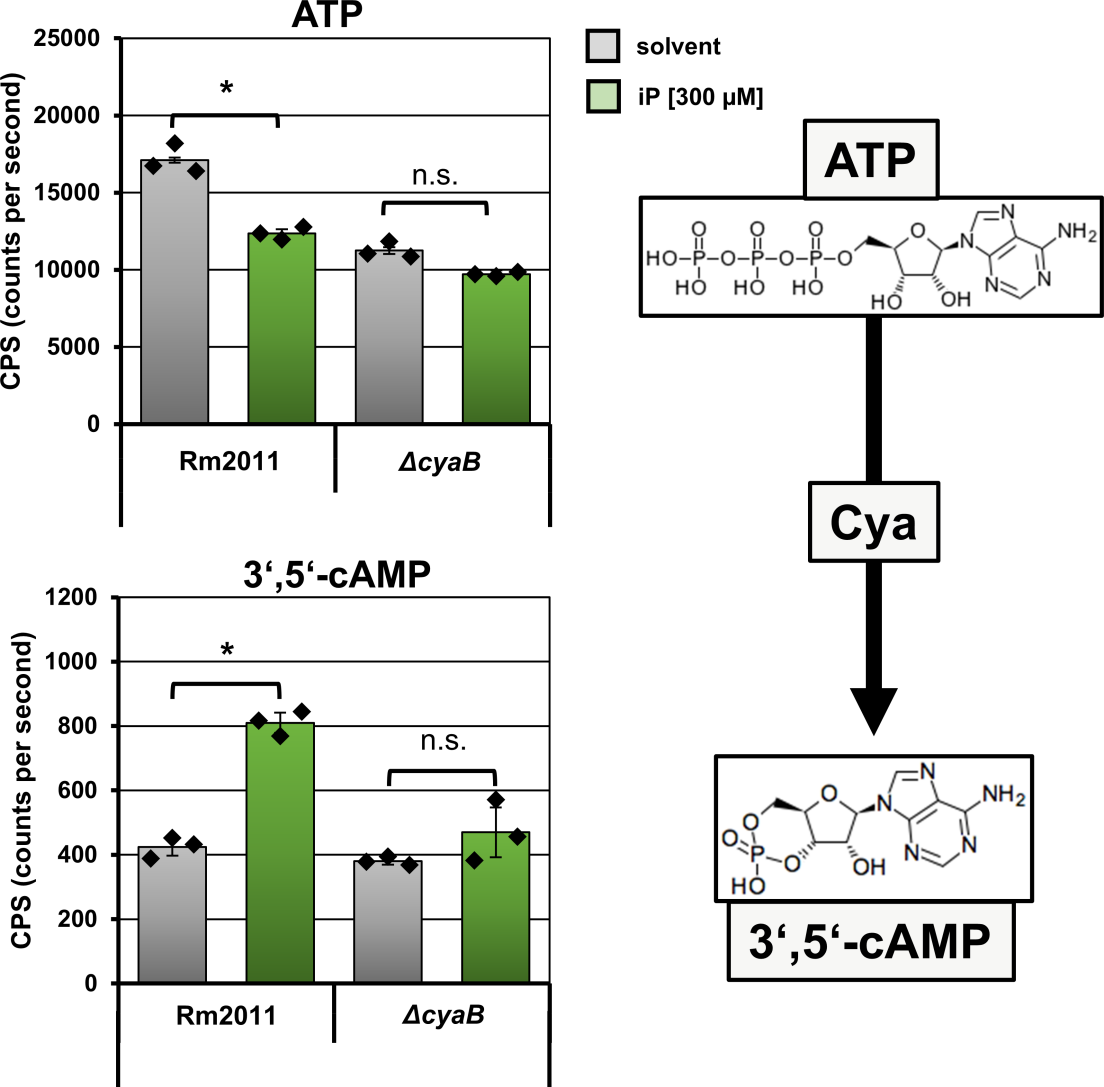
*S. meliloti* Rm2011 responds to iP with CyaB-mediated increase in intracellular cAMP levels, accompanied by a decrease in intracellular ATP levels. LC-MS/MS analysis of intracellular 3′,5′-cAMP and ATP in Rm2011 and *ΔcyaB* strains. Metabolites were extracted from cells grown for 24 h in Vincent minimal medium supplemented with either solvent only or 300 µM iP. Error bars represent the standard deviation of three technical replicates, and the diamonds indicate the single values. Asterisks indicate significance of the differences determined in Student’s *t*-test. (*): *P* < 0.05, n.s.: not significant.

To determine the effect of iP on intracellular cAMP levels, we employed targeted mass spectrometry analysis. In Rm2011 wild-type cells induced with 300 µM iP, we observed an approximately twofold increase in intracellular cAMP levels compared to either the control wild-type cells (no iP added) or *∆cyaB*-deficient cells (Fig. 2). Furthermore, a decrease in ATP levels in the iP-treated wild-type cells correlated with the increase in cAMP, which may be partly due to enhanced ATP consumption by cAMP synthesis. Taken together, these data strongly suggest that *S. meliloti* is able to respond to plant CKs with cAMP biosynthesis by CyaB.

### The ability of CyaB to respond to iP is conserved within *Rhizobiaceae*

**Fig 3:**
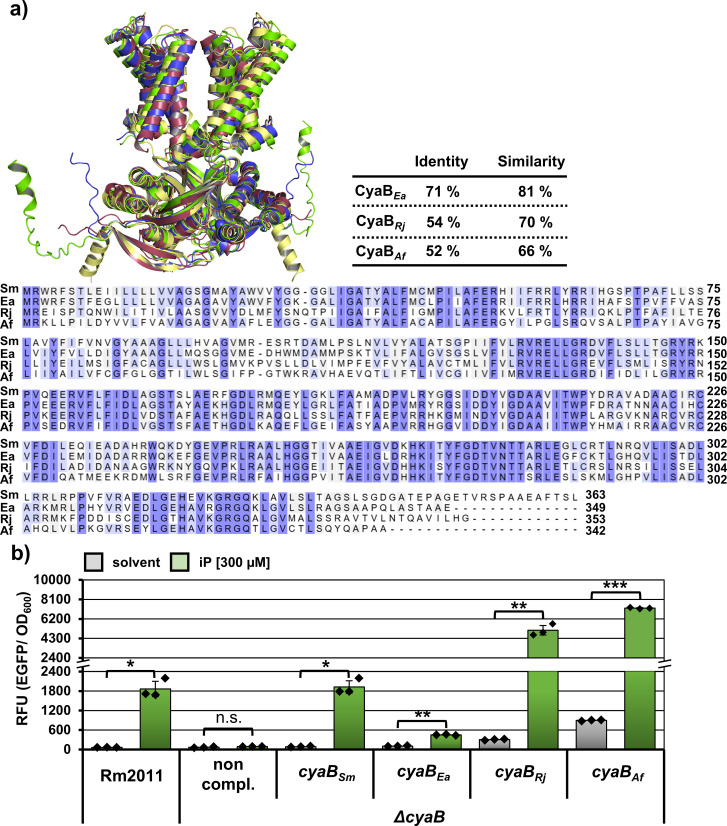
Complementation of the *S. meliloti cyaB* deletion with *cyaB* orthologs from other species of the *Rhizobiaceae*. (**a**) Upper panel, left side: superimposition of CyaB protein dimer structures predicted with the AlphaFold3 algorithm. Green, CyaB*_Sm_*; blue, CyaB*_Ea_*; yellow, CyaB*_Rj_*; red, CyaB*_Af_*. Upper panel, right side: amino acid sequence homology of the selected CyaB orthologs to *S. meliloti* CyaB. The numbers indicate the protein identity and similarity (Needleman-Wunsch algorithm) to CyaB*_Sm_*. Lower panel: multiple sequence alignment of amino acid sequences of CyaB*_Sm_*, CyaB*_Ea_*, CyaB*_Rj_,* and CyaB*_Af_* utilizing the Clustal Omega algorithm (UniProt). Dark blue coloring indicates amino acid similarity between all four sequences; light blue indicates similarity between three sequences. (**b**) Reporter fluorescence of the Rm2011 wild type, the *∆cyaB* strain alone (non-compl.) and *∆cyaB* strain complemented with *cyaB*_Sm_, *cyaB*_Ea_, *cyaB*_Rj_, or *cyaB*_Af_. The strains carried the reporter plasmid pSRKKm-smc02178-EGFP and were grown for 24 h in Vincent minimal medium supplemented with either solvent or 300 µM iP. The error bars represent the standard deviation of three biological replicates, and the diamonds show the single values. Asterisks indicate significance of the differences determined in Student’s *t*-test. (***): *P* < 0.0005, (**): *P* < 0.005, (*): *P* < 0.05, n.s.: not significant.

The family *Rhizobiaceae* comprises bacteria with different lifestyles ranging from free-living to pathogenic or symbiotic. We asked whether the CyaB-mediated plant CK response is conserved within the *Rhizobiaceae* and if it is correlated with the plant-associated lifestyle. Therefore, we selected *A. fabrum* (formerly *A. tumefaciens*), *Rhizobium johnstonii* (formerly *Rhizobium leguminosarum*), and *Ensifer adhaerens* (34–36) as representative *Rhizobiaceae* species with plant-pathogenic, plant-symbiotic, and non-plant-associated lifestyles, respectively. CyaB was well conserved in the respective genera (Fig. 3a; Fig. S1). To test whether the CyaB orthologs from the chosen species were capable of responding to iP, we introduced *cyaB_Af_* (Atu1149), *cyaB_Rj_* (RL1602), and *cyaB_Ea_* (FA04_04595) into the *S. meliloti ∆cyaB* strain by double homologous recombination. The orthologous genes were inserted into the native *cyaB* locus under the control of the native *S. meliloti cyaB* promoter. The resulting strains were analyzed for their ability to respond to iP using the *smc02178-EGFP* reporter. This analysis revealed a strong increase of EGFP fluorescence in iP-treated *∆cyaB* cells producing CyaB*_Af_* and CyaB*_Rj_* but only a moderate increase in the CyaB*_Ea_*-producing strain (Fig. 3b). However, the reporter activity promoted by the respective CyaB orthologs in the absence of iP also differed, with the lowest EGFP fluorescence conferred by CyaB*_Ea_* (Fig. 3b). This may indicate differences in overall enzymatic capacity or protein stability between the tested CyaB orthologs.

### iP signal perception is not mediated by *cyaB-*adjacent chromosomal genes, genes located on pSymA, or CHASE domain containing cyclic-di-GMP synthases

CyaB does not contain any known sensory domain that could obviously account for iP signal perception. It solely contains an N-terminal transmembrane domain composed of four transmembrane helices and a C-terminal cytoplasmic catalytic ACYC domain. Therefore, we used genetic analyses to test potential additional auxiliary factors, which may be required for the iP signal perception by CyaB.

Since the *S. meliloti* megaplasmid pSymA carries genes essential for symbiosis, we asked if it carries an iP signaling co-factor gene. The iP response of the *S. meliloti* Rm2011 derivative strain RMP3498c lacking pSymA was similar to that of the isogenic wild type-like strain RMP3499c carrying pSymA (Fig. S2). This result rules out that genes on pSymA are required for the iP response.

In plants, CK sensing is known to be mediated by CHASE domain-containing proteins (37, 38). *S. meliloti* Rm2011 possesses a total of five CHASE domain family proteins. Three of those are the CHASE2 domain AC/GCs CyaD1, CyaD2, and CyaK, whose role in the iP response has already been ruled out (Fig. 1d). The other two proteins are the diguanylate cyclase/phosphodiesterases Smc03178 and Sma0137, containing a CHASE and a CHASE4 domain, respectively. Therefore, we tested the iP response in *S. meliloti* strain Rm2011 ∆XVI, lacking all the diguanylate cyclase genes including *smc03178* and *sma0137* (Fig. S2). Since components of the same signaling pathway are often encoded in close proximity, we also asked if genes surrounding *cyaB* on the *S. meliloti* Rm2011 chromosome could encode a CyaB co-factor mediating the iP perception. Therefore, we generated deletion mutants in six *cyaB*-proximal genes and tested the iP response. Neither the loss of the CHASE-domain family diguanylate cyclases/phosphodiesterases nor gene knockouts in the *cyaB*-surrounding genes abolished the iP-mediated EGFP fluorescence response, conferred by the *smc02178-EGFP* reporter (Fig. S2 and S3). Interestingly, an approximately 1.8-fold reduction in the EGFP fluorescence was observed upon deletion of the *smc01754* gene, located upstream of *cyaB* and encoding a multicopper oxidase.

### CyaB and its orthologs interact with the MEP/DOXP pathway protein Dxs

To directly identify potential interaction partners of CyaB, we used CoIP, coupled to protein identification by mass spectrometry. CyaB, C-terminally fused to the 3xFLAG tag (CyaB-C3F), was produced in the *∆cyaB* strain from the mid-copy vector pSRKKm under the control of the native *cyaB* promoter. The empty vector pSRKKm-3xFLAG was used as a negative control. Cells carrying the respective plasmids were grown in Vincent minimal medium for 24 h with or without the addition of 300 µM iP and processed as described in the Material and Methods section.

**Fig 4:**
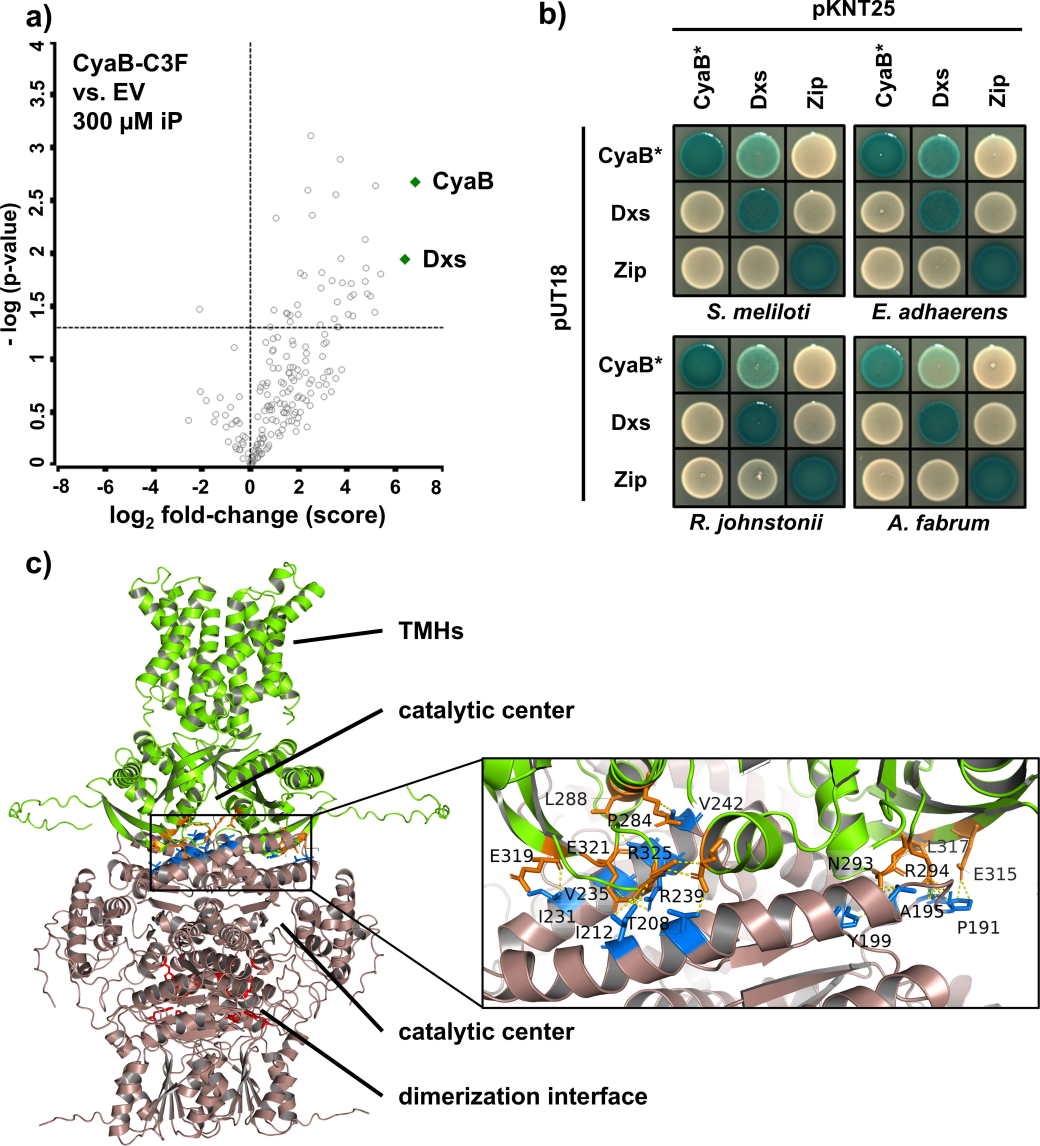
Identification and validation of CyaB-Dxs interaction. (**a**) Proteins identified in the CoIP samples of the *ΔcyaB* strain producing CyaB-3xFLAG (CyaB-C3F) as a bait or carrying an empty vector pSRKKm-3xFLAG (EV). Cells were grown for 24 h in Vincent minimal medium supplemented with 300 µM iP. Volcano plot showing a two-sample test of CyaB-C3F vs EV samples. The data points represent the mean of three biological replicates. Vertical dotted line: log_2_ fold change of protein abundance; horizontal dotted line: significance (−log(0.05) = 1.3). (**b**) Bacterial two-hybrid analysis of interactions between *S. meliloti* Dxs and enzymatically inactive CyaB variants (CyaB*) from *S. meliloti*, *R. jonstonii*, *A. fabrum*, or *E. adhaerens*. Four independent co-transformant colonies were analyzed, and the representative result is shown. Blue staining indicates protein-protein interactions. (**c**) Alphafold3 prediction of the protein-protein interaction between dimers of CyaB (green) and Dxs (beige). The potentially interacting amino acids (distance range < 3 Å) of CyaB and Dxs are shown in orange and blue, respectively. The Dxs dimerization interface (according to reference 43) is highlighted in red.

In both growth conditions, the cytoplasmic 1-deoxyxylulose-5-phosphate (DXP) synthase Dxs was reliably identified as the top-ranked protein in CyaB-C3F samples but not in the negative control samples (Fig. 4a; Table S1 and S2). Dxs synthesizes DXP from D-glyceraldehyde 3-phosphate (G3P) and pyruvate, and the corresponding gene is essential in *Escherichia coli* and *S. meliloti* (39, 40). DXP is a crucial precursor for the biosynthesis of thiamine, pyridoxine, and terpenoid backbone in the MEP/DOXP pathway (41, 42).

**Fig 5:**
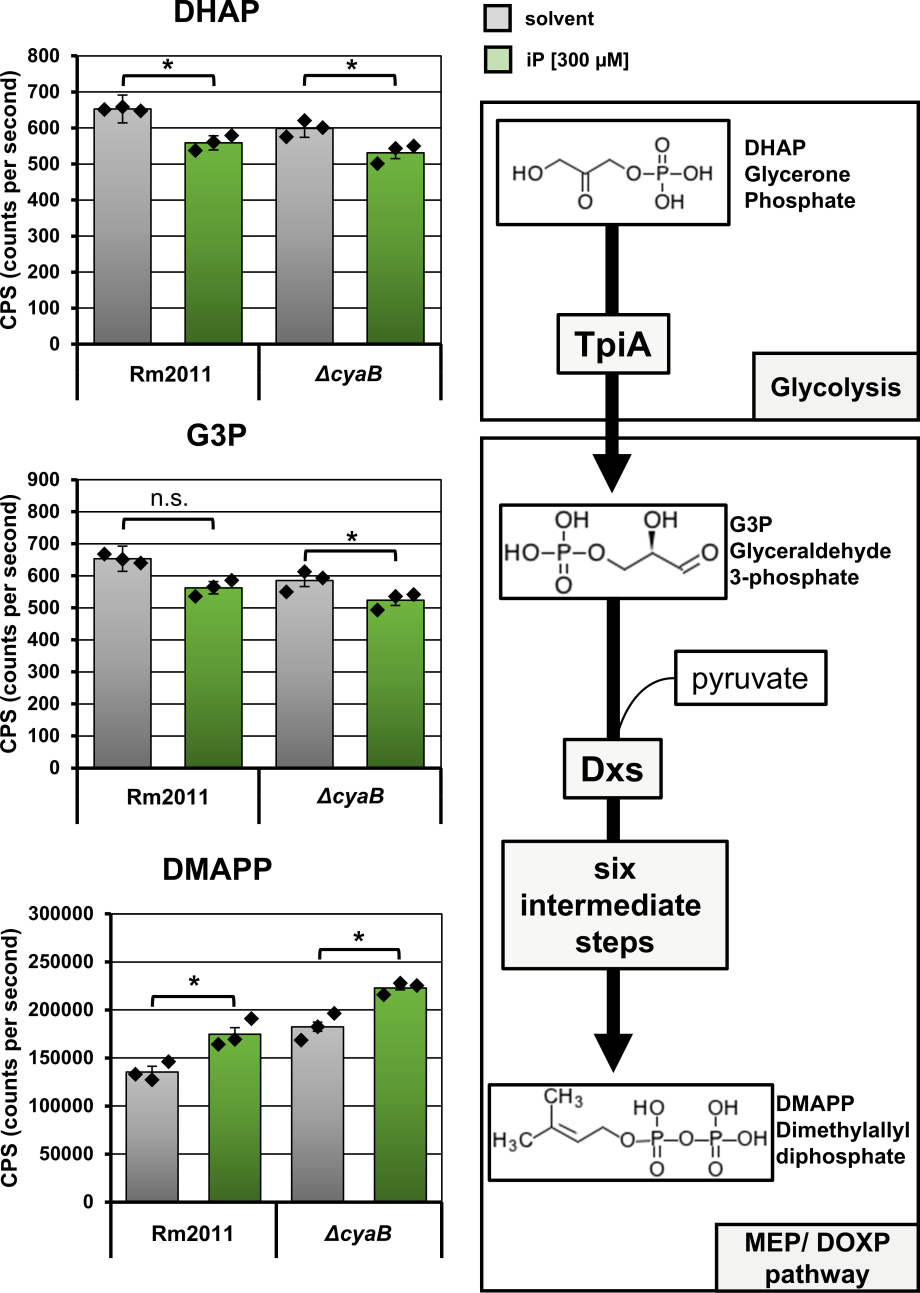
*S. meliloti* Rm2011 responds to iP with alterations in intracellular levels of glycolysis and MEP/DOXP pathway metabolites. The left panel shows the relative abundance of the indicated metabolites determined by LC-MS/MS analysis. Metabolites were extracted from cells grown for 24 h in Vincent minimal medium supplemented with either solvent or 300 µM iP. Error bars represent the standard deviation of three technical replicates, and the diamonds indicate the single values. Asterisks indicate significance of the differences determined in Student’s *t*-test. (*): *P* < 0.05, n.s.: not significant. The right panel shows a simplified scheme of the glycolytic DHAP-G3P conversion catalyzed by triosephosphate isomerase TpiA (SMc01023) and the downstream synthesis of DMAPP in the MEP/DOXP pathway.

To evaluate the possible effect of iP and CyaB on Dxs activity, we quantified the DXP precursor G3P, the G3P precursor glycerone phosphate (DHAP), and dimethylallyl diphosphate (DMAPP) as a downstream biosynthetic product in the wild type and △*cyaB* cells, grown in Vincent minimal medium with or without the addition of 300 µM iP. This analysis revealed a minor decrease of DHAP and G3P levels in iP-treated wild-type and *∆cyaB* cells compared to the untreated controls (Fig. 5). In contrast, DMAPP increased by more than 20% in cells grown with iP. Notably, deletion of *cyaB* alone resulted in approximately 30% increase in DMAPP levels (Fig. 5). Taken together, these results might hint at Dxs activity modulation by iP and CyaB.

To validate the CoIP result, we tested the protein-protein interaction between CyaB and Dxs in the bacterial two-hybrid (BTH) system based on the reconstitution of *Bordetella pertussis* AC from fragments T25 and T18, each fused to one of the proteins of interest. The fusion proteins are produced in the cAMP-deficient *E. coli* BTH101 strain, and if protein-protein interaction occurs, the β-galactosidase gene is activated by CAP-cAMP, which results in a blue staining on agar medium plates with 5-bromo-4-chloro-3-indolyl-β-D-galactopyranoside (X-Gal) (44). To avoid cross-activation of the system due to AC activity of CyaB, we generated the variant CyaB*_Sm_, carrying amino acid substitutions D162H, G205A, and D206H. The corresponding residues are predicted to mediate the binding of the metal co-factor and ATP in the active site and therefore are supposed to be required for the AC activity. The T25- and T18-fragments were C-terminally fused to CyaB*_Sm_ and Dxs to ensure cytoplasmic localization of the fusion proteins.

The BTH analysis revealed self-interaction of both CyaB*_Sm_ and Dxs, as well as Dxs-CyaB*_Sm_ interaction involving Dxs-T25 and CyaB*_Sm_-T18 (Fig. 4b). The negative control combination of CyaB*_Sm_-T18 and T25-Zip showed no staining, confirming the absence of AC activity of CyaB*_Sm_ and the genuineness of the observed interaction. The negative result obtained with the Dxs-T18 and CyaB*_Sm_-T25 combination may be explained by the lower copy number of the pKT25 plasmid compared to the pUT18 plasmid, which might result in lower levels of CyaB*_Sm_ protein, insufficient to detect the interaction.

Since CyaB from *A. fabrum*, *R. johnstonii*, and *E. adhaerens* were able to substitute for the absence of CyaB_Sm_ regarding the iP-induced activation of the cAMP-responsive fluorescent reporter, we asked if these proteins were also able to interact with Dxs. The Dxs protein is highly conserved in these species, with amino acid sequence similarity to *S. meliloti* Dxs greater than 90%. Thus, we tested for interactions of the CyaB orthologs with *S. meliloti* Dxs in BTH. The corresponding genes were subjected to site-directed mutagenesis to obtain the AC-deficient variants cyaB*_Af_, cyaB*_Rj_, and cyaB*_Ea_. CyaB*_Af_ and CyaB*_Ea_ were mutated at the same positions as CyaB*_Sm_, while in CyaB*_Rj_, substitutions D164H, G207A, and D208H were introduced. All three CyaB* orthologs showed self-interaction and interaction with Dxs, similar to CyaB*_Sm_ (Fig. 4b). Taken together, these data suggest that CyaB is capable of interacting with Dxs and that this feature is conserved within the *Rhizobiaceae*.

The structure of the protein complex between dimers of *S. meliloti* Dxs and CyaB was predicted using the Alphafold3 algorithm. The resulting model (Fig. 4c) revealed multiple potential Dxs-CyaB contacts at the residues A190-E317, A190-R294, P191-R294, P191-E315, A195-N293, Y199-N293, T208-E171, I212-R325, I231-E319, V235-E321, A238-L288, R239-E321, and V242-R284. The Dxs-CyaB interface was adjacent to the CyaB active site. CyaB residue R284, potentially interacting with V242 of Dxs, is a part of the CyaB active site, since it belongs to the conserved nucleotidyl binding site motif of the class III nucleotidyl cyclases. This observation aligns with the proposed role of Dxs in regulation of CyaB enzymatic activity and opens a new research direction for investigation of the underlying molecular mechanisms.

## DISCUSSION

The CK signaling plays an important role in many aspects of plant development. In *M. truncatula*, CK synthesis gene expression and the concomitant CK response are activated in the nodule primordia (45), and an exposure to rhizobial-derived nodulation factors mediates an increase in iP levels in *M. truncatula* roots (23). Thus, the plant responds to the rhizobial symbiont with CK signaling, which in turn may provide a regulatory cue for the rhizobial symbiont. On the other hand, *S. meliloti* and related rhizobia are able to produce CKs and to release them into the growth medium (31, 32). Under experimental conditions, external application of CKs triggers the plant nodule development program even in the absence of the bacterial symbiont (17, 46). However, the CKs produced by cultured rhizobia failed to exert such an effect, likely due to very low production levels (31, 32).

In the early stages of the *S. meliloti-Medicago* symbiosis, cAMP signaling plays an important role in maintaining the optimal infection rate via repression of secondary infections (6, 12). In this study, we uncovered a novel mechanism of sensing plant-derived compounds by *S. meliloti*. Although the role of CyaB-mediated CK perception *in planta* remains to be demonstrated, activation of cAMP synthesis in response to the CK phytohormones constitutes an additional sensory pathway for symbiotic molecular communication. It may either work in concert with the activation of CHASE2 domain AC/GCs by phytochemicals of protein nature (9, 10), or represent an independent regulatory pathway, depending on the spatiotemporal pattern of the plant signals that activate CyaB or the CHASE2 domain AC/GCs during symbiotic nodule development.

tZ is structurally very similar to iP, except for an additional hydroxyl group on the isopentenyl side chain. The biological activity of iP and tZ in *S. meliloti* differed substantially, as reflected by an approx. 10-fold stronger reporter response to iP. This difference could be explained either by unequal molecular stability or biological availability of the two CKs, or by specific structural features of the receptor that confer this specificity. To date, the only CK receptor reported in bacteria is the *X. campestris* CHASE domain histidine kinase PcrK. PcrK responded robustly with autokinase activity to iP, but only weakly to tZ, kinetin, or cis-zeatin (30). This iP specificity of PcrK has been attributed to its structural properties (29), suggesting that bacteria need and are able to distinguish between plant CKs, even if they are structurally very similar. In plants, tZ is mainly synthesized in the roots and is transported to the shoots via the xylem, whereas iP has been proposed to be a shoot CK, transported to the roots via the phloem (47). In *L. japonicus*, shoot iP levels increased in response to symbiotic root infection, and the shoot-derived CKs were shown to modulate the nodule number, thereby participating in the autoregulation of nodulation (26). Therefore, it is tempting to speculate that the observed difference in cAMP response to either iP or tZ is related to the particular biological roles or localization of the two CKs in the host plant.

The ability of CyaB to activate the cAMP reporter in response to iP and to interact with Dxs was conserved in the analyzed CyaB orthologs from *A. fabrum*, *R. johnstonii*, and *E. adhaerens*. However, the reporter activity in *S. meliloti* △*cyaB* strain, complemented with the respective CyaB orthologs, differed. The strongest reporter response was observed with CyaB_Af_ derived from a plant pathogen and the weakest with CyaB_Ea_ derived from a free-living species (Fig. 3). We cannot exclude that the analyzed CyaB homologs differed in their enzymatic capacity or protein levels, resulting in different strengths of the iP response. On the other hand, the ability to promote strong cAMP-mediated reporter activation in the presence of iP correlated with the plant-associated lifestyle of the CyaB source organism, at least in the small number of species considered in this study. This leads us to hypothesize a possible evolutionary adaptation of CyaB to plant signal perception, which requires further investigation.

**Fig 6:**
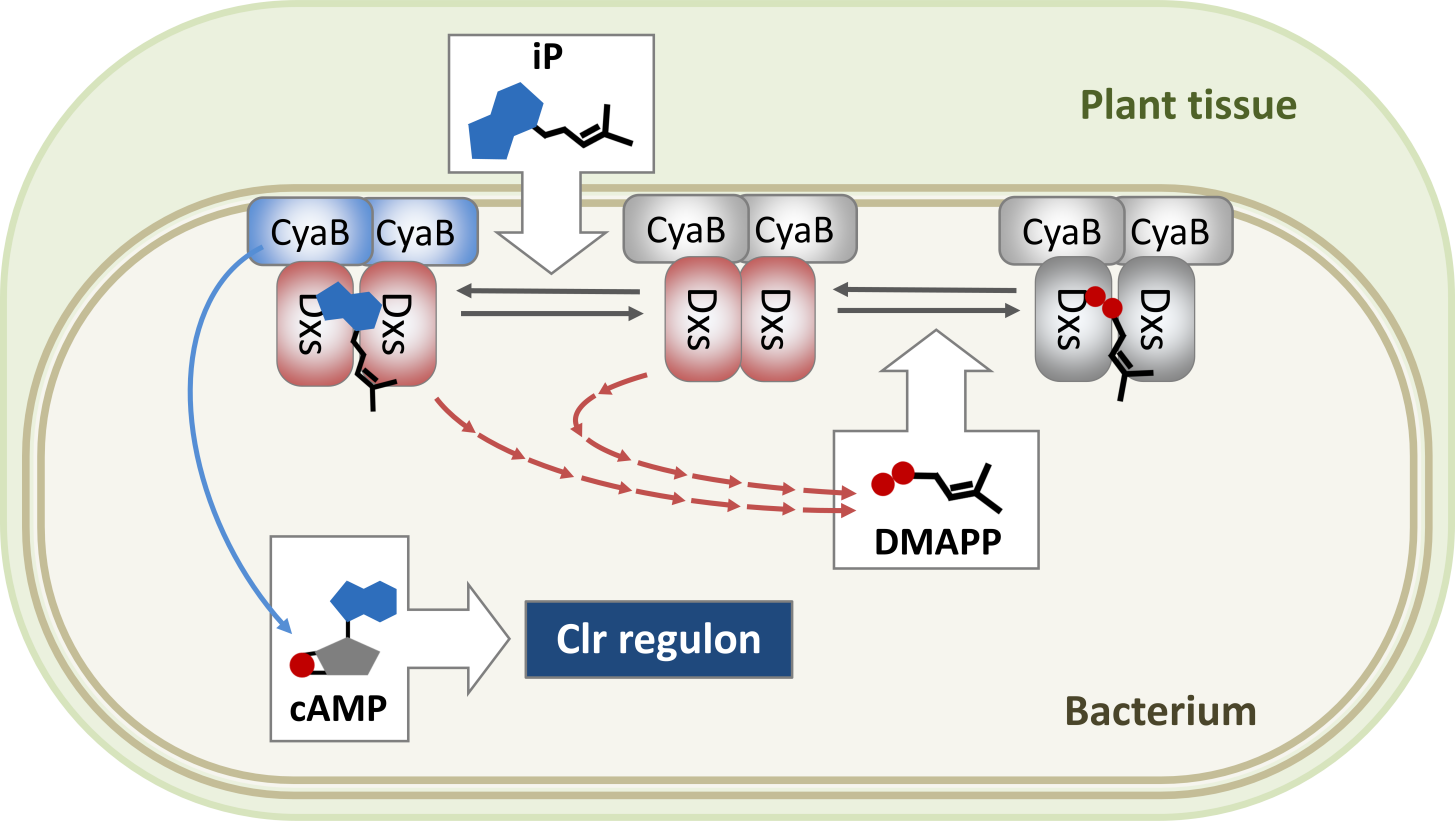
Hypothetical model of iP-mediated regulation of CyaB. Dxs forms a complex with CyaB. Binding of plant metabolite iP to Dxs is suggested to activate cAMP synthesis by CyaB and, subsequently, of the Clr regulon. Dxs is subject to feedback inhibition by the downstream biosynthesis product DMAPP. iP might compete with DMAPP for Dxs binding. Active enzymes are colored and inactive are shown in gray.

Our results show that CyaB interacts with Dxs both in the presence and the absence of iP. Dxs catalyzes synthesis of DXP from G3P and pyruvate, providing a crucial precursor for the thiamine, pyridoxine, and terpenoid backbones in the MEP/DOXP biosynthetic pathways (41, 42). Dxs is active as a dimer and requires the thiamine diphosphate (TPP) co-factor. The feedback inhibition by the downstream products of the terpenoid biosynthetic pathway DMAPP and isopentenyl diphosphate (IPP) results in monomerization, aggregation, and ultimately degradation of Dxs (43). It was originally proposed that DMAPP and IPP compete with TPP for the Dxs binding (48). This model was recently challenged by a new study that introduced an allosteric inhibition mechanism, where DMAPP and IPP interfere with Dxs dimerization through binding at the protein-protein interaction interface (43). Our metabolite analyses identified slightly elevated DMAPP levels in iP-treated *S. meliloti* cells. Since the side chain of iP is identical to that of DMAPP, it is tempting to speculate that iP can compete with DMAPP for the Dxs binding site, thereby relieving the feedback inhibition. Furthermore, the mere absence of CyaB caused a slight increase in DMAPP levels, raising the possibility that the CyaB-Dxs interaction could influence Dxs function. The molecular mechanism of CyaB activation by iP remains unclear. However, the data presented in this work provide a hint that it could involve iP binding by Dxs, resulting in a conformation change of its interaction partner CyaB, favoring the CyaB AC activity (Fig. 6).

## MATERIALS AND METHODS

### Strains, plasmids, and growth conditions

Strains and plasmids used in this study are listed in Table S3. For cloning and conjugation, *E. coli* was cultured in Lysogeny broth (LB, tryptone 10 g/L, yeast extract 5 g/L, NaCl 5 g/L) media at 37°C. For agar plates, 15 g/L agar was added to the media prior to autoclaving. Antibiotics were added to the media at the following concentrations (liquid/ agar plates): kanamycin 50/100 mg/L, chloramphenicol 10/20 mg/L, ampicillin 100/150 mg/L, and spectinomycin 50/100 mg/L.

*S. meliloti* was cultured in TY (tryptone, 5 g/L; yeast extract, 3 g/L; and CaCl_2_*2H_2_O, 0.4 g/L) or Vincent minimal media (K_2_HPO_4_ 14.7 mM, KH_2_PO_4_ 11.5 mM, MgSO_4_ 1 mM, mannitol 55 mM, Na-glutamate 5.5 mM, CaCl_2_ 0.5 mM, FeCl_3_ 0.037 mM, biotin 4.1 µM, H_3_BO_3_ 3 mg/L, MnSO_4_*4H_2_O 2.23 mg/L, ZnSO_4_*7H_2_O 0.287 mg/L, CuSO_4_*5H_2_O 0.125 mg/L, CoCl_2_*6H_2_O 0.065 mg/L, and NaMoO_4_*2H_2_O 0.12 mg/L) at 30°C. For agar plates, 15 g/L agar was added to the media prior to autoclaving. Antibiotics were added to the media at the following concentrations (liquid/agar plates): kanamycin 100/200 mg/L, streptomycin 300/600 mg/L, and spectinomycin 100/200 mg/L.

CKs (N6-(∆2-isopentenyl) adenine [iP], Biomol GmbH; tZ, Sigma-Aldrich; 6-BAP, Sigma-Aldrich; kinetin, Sigma-Aldrich) and the auxin compound IAA (Sigma-Aldrich) were dissolved in DMSO (measurements shown on Fig. 1a) or ethanol (all the other measurements). 3′, 5′-cAMP (cAMP, Biolog Life Science) was dissolved in H_2_O. To achieve the indicated final CK and auxin concentrations, 1.5 µL of CK or auxin solution was added to 100 µL of the culture. For the negative control (solvent), 1.5 µL of DMSO (measurements shown on Fig. 1a) or ethanol (all the other measurements) was added to 100 µL of the culture.

### Cloning and genetic manipulations

The constructs used in this study were generated using standard genetic procedures. All the constructs were verified by Sanger sequencing. Oligonucleotides used are listed in Table S4.

Plasmids were transferred into the indicated *S. meliloti* strain via triparental mating using the *E. coli* DH5α strain harboring the plasmid and the *E. coli* MT616 helper strain (49). For chromosomal gene deletion or integration, the suicide pK18mobsacB constructs, harboring the desired gene deletion or gene flanked by two homologous regions (500 bp), were introduced into the corresponding *S. meliloti* strain by conjugation. Strains with chromosomally integrated pK18mobsacB constructs were selected on TY plates with kanamycin and streptomycin. The transconjugants were grown overnight in TY medium without antibiotics and plated on LB agar containing 10% sucrose for selection of the plasmid backbone loss due to a double homologous recombination (50). The resulting colonies were tested for the absence of kanamycin resistance, and the gene integration or deletion was confirmed by PCR.

To generate catalytically inactive CyaB variants, point mutations in the respective genes were introduced using overlap extension PCR.

### Fluorescence measurements

Fluorescence measurements were performed using the respective transconjugants harboring the indicated reporter constructs. Precultures were grown in 100 µL TY medium supplemented with kanamycin, overnight at 30°C with shaking at 1,200 rpm in 96-well polystyrene flat-bottom plates (Greiner). Replicate precultures were inoculated with three to four single colonies of the given transconjugant strain. One microliter of the precultures was used to inoculate the cultures in 100 µL Vincent minimal medium supplemented with kanamycin and either the solvent or the indicated test substances. The cultures were grown in 96-well plates for 24 h at 30°C with shaking at 1,200 rpm. EGFP fluorescence measurements were carried out using a Tecan Infinite 200 PRO multimode reader (fluorescence: excitation, 488 nm; emission, 522 nm; optical density: absorbance 600 nm). The relative fluorescence unit (RFU) was calculated by dividing the fluorescence value by the optical density. The background fluorescence of the corresponding strains containing the empty vector pSRK-EGFP was subtracted from the RFU values.

### Flow cytometry measurements

Replicate precultures were inoculated with three to four independent *S. meliloti* pSRKKm-smc02178-EGFP transconjugant colonies and grown for 24 h at 30°C in 3 ml TY supplemented with kanamycin. Thirty microliters of a preculture was used to inoculate the main cultures in 3 mL Vincent minimal medium containing kanamycin and either the solvent or 300 µM iP. The cultures were grown for 24 h at 30°C. Flow-cytometry-assisted fluorescence measurements were performed using a BD Fortessa Flow Cytometer (BD Biosciences, Germany) with a 488 nM excitation laser. The data analysis was performed as previously described, considering at least 28,000 events per sample (51).

### Protein-protein interaction analysis by CoIP and mass spectrometry

CoIP experiments and protein detection via mass spectrometry were performed as previously described (52). Briefly, the *S. meliloti ∆cyaB* strain producing CyaB-C3F and the negative control strain carrying plasmid pSRK-3xFLAG were grown in 200 mL of TY medium, containing either the solvent or 300 µM iP, to an OD_600_ of 0.4 to 0.6. The proteins were cross-linked by adding 0.36% formaldehyde to the cultures and incubating for 15 min at room temperature (RT). The cultures were then quenched for 10 min with 0.35 M glycine and washed once with 25 mL ice-cold TE buffer (10 mM Tris, 1 mM EDTA, pH 8.0) containing 0.1% lauryl sarcosinate and twice with 50 mL ice-cold phosphate-buffered saline (1.8 mM KH_2_PO4, 8 mM Na_2_HPO4, 2.7 mM KCl, and 147 mM NaCl, pH 7.2). Cell pellets were resuspended in 4 mL ice-cold lysis buffer (50 mM Tris-HCl pH 7.4, 150 mM NaCl, 1 mM EDTA, 1% Triton X100, and 1 mM PMSF) and lysed using a French press instrument. Cell debris was pelleted by centrifugation at 30,000 × *g* for 30 min at 4°C. The cleared lysates were incubated with anti-FLAG M2 affinity gel (FLAG immunoprecipitation kit; Sigma), and proteins were purified according to the manufacturer’s manual. Bound proteins were eluted with FLAG peptide solution and submitted for mass spectrometry measurements, which were performed as described previously (53). Proteins detected with two or more peptides in every replicate were considered for the further analysis (Table S5). The Score Sequest HT values were used for ranking. The volcano plot was generated using two-sample Student’s *t*-test with Perseus V1.6.15.0 software (54).

### Bacterial two-hybrid analysis

Bacterial two-hybrid experiments were performed as previously described (44). Briefly, *E. coli* BTH101 was co-transformed with corresponding pUT18Spe and pKNT25Spe plasmid pairs. Single co-transformant colonies were used to inoculate 100 µL LB, supplemented with ampicillin and kanamycin, in a 96-well plate and incubated at 37°C for 4 h. Ten microliters of each culture was spotted on LB agar plates containing ampicillin, kanamycin, 100 mg/L X-Gal, and 500 µM isopropyl-βbeta𝛽-d-thiogalactopyranosid (IPTG). Plates were incubated for 24 h at 30°C and afterwards for 24 h at RT in the dark prior to imaging. For each co-transformation, four independent colonies were analyzed.

### Targeted metabolome measurements

Fifty milliliters of Vincent minimal medium, supplemented with either 300 µM iP or the solvent as a negative control, was inoculated with the *S. meliloti* Rm2011 wild type or *∆cyaB* strain to an OD_600_ of 0.2. After 24 h of growth at 30°C, 5 mL of the culture were mixed with 5 mL quenching solution (70% MeOH, −80°C), distributed in 2 mL reaction tubes, and pelleted for 10 min at 13,000 × *g* and −10°C. The pellets were stored at −80°C until further use.

For metabolite extraction, pellets were resolubilized in 200 µL of extraction buffer (50% MeOH, 50% TE-buffer [10 mM TRIZMA, 1 mM EDTA, pH 7.0], stored for 24 h at −20°C), and subsequently the same amount of chloroform was added (stored for 24 h at −20°C). The mixture was incubated for 2 h at 4°C and shaken at 1,000 rpm. The extraction mixture was centrifuged at −10°C for 10 min at 20,000 × *g*. The aqueous phase was filtered using a hydrophobic polytetrafluorethylen (PTFE) filter (0.2 µm pore size, 4 mm diameter, Millipore) and stored at −80°C until analysis.

Qualitative determination of metabolites was performed using a liquid chromatography-tandem mass spectrometry (LC-MS/MS). The chromatographic separation was performed on an Agilent Infinity II 1290 HPLC system using a SeQuant ZIC-pHILIC column (150 × 2.1 mm, 5 μm particle size, peek coated, Merck) connected to a guard column of similar specificity (20 × 2.1 mm, 5 μm particle size, Phenomenex). A constant flow rate of 0.1 mL/min was applied at 40°C using mobile phase A (10 mM ammonium acetate in water, pH 9, supplemented with medronic acid to a final concentration of 5 μM) and mobile phase B (10 mM ammonium acetate in 90:10 acetonitrile to water, pH 9, supplemented with medronic acid to a final concentration of 5 μM). The injection volume was 2 µL. The mobile phase profile consisted of the following steps and linear gradients: 0–1 min constant at 75% B; 1–6 min from 75 to 40% B; 6–9 min constant at 40% B; 9–9.1 min from 40 to 75% B; and 9.1–20 min constant at 75% B. An Agilent 6495 ion funnel mass spectrometer was used in negative and positive ionization mode with an electrospray ionization source and the following conditions: ESI spray voltage 3,500 V, nozzle voltage 1,000 V, sheath gas 300°C at 9 L/min, nebulizer pressure 20 psig, and drying gas 100°C at 11 L/min. Compounds were identified based on their mass transition and retention time compared to standards. Chromatograms were integrated using MassHunter software (Agilent, Santa Clara, CA, USA). Relative metabolite abundance was determined based on signal intensity. Counts per second value was calculated by dividing the relative metabolite signal intensity by the OD_600_ value.

Mass transitions, collision energies, cell accelerator voltages, and dwell times have been optimized using chemically pure standards (Table 1).

**TABLE 1:** Optimized parameter settings of the analyzed metabolites

Name	Precursor ion	Product ion	Collision energy (V)	Fragmentor voltage (V)	Cell accelerator voltage (V)	Dwell time (msec)	Polarity
ATP	505.9	407.9 158.8	21 28	380	5	65	Negative
cAMP	328	134.1 79	25 36	380	5	65	Negative
DMAPP	244.9	244.9 79.1	0 31	380	5	65	Negative
GAP/DHAP	169	97 79	5 50	380	5	65	Negative

### Phylogenetic tree generation and *in silico* analysis

To assess the phylogenetic relationship of CyaB orthologs, a BLASTP search within the Kyoto Encyclopedia of Genes and Genomes database was performed using the BLOSUM62 matrix and *E*-value threshold of 1^e−40^. Sequences (cutoff 280 bits) were selected and filtered for unique species (different strains were considered as one species; best hit of each species was chosen for analysis). The phylogenetic tree was generated via the NGPhylogeny online tool (https://ngphylogeny.fr/) using the default parameters (“One Click”). The resulting phylogenetic tree (Newick format) was uploaded to iTOL for optical readjustments (https://itol.embl.de/).

For protein structure prediction, the web-based AlphaFold3 algorithm was used (https://alphafoldserver.com/). The full-length protein sequence was used for the prediction. The proteins were predicted in a dimeric form. Multiple sequence alignment of full-length protein sequences was performed using the Clustal Omega algorithm (http://www.clustal.org/) via the web-based function of the UniProt web page suite (https://www.uniprot.org/align).

## SUPPLEMENTAL MATERIAL

Supplemental material jb.00585-25-s0001.pdf Figures S1 to S3 and Tables S1 to S4.

Table S5 jb.00585-25-s0002.xlsx Mass spectrometry data.

ASM does not own the copyrights to Supplemental Material that may be linked to, or accessed through, an article. The authors have granted ASM a non-exclusive, world-wide license to publish the Supplemental Material files. Please contact the corresponding author directly for reuse.
