# Supplementary material for: CHASE-independent cytokinin perception triggers 3′,5′-cAMP signaling in Sinorhizobium meliloti
Source: J Bacteriol. 2026 Feb 25;208(3):e00585-25. doi: 10.1128/jb.00585-25 (PMC13001267; doi:10.1128/jb.00585-25)
Supplement: Supplemental material — Figures S1 to S3 and Tables S1 to S4. [file jb.00585-25-s0001.pdf]

Supplemental material

CHASE-independent cytokinin perception triggers 3’,5’-cAMP signaling in *Sinorhizobium meliloti*

Niklas M. Schäfer[a], Elizaveta Krol[a], Nicole Paczia[b], Neda Farmani[a], Anke Becker[a#]

[a] Center for Synthetic Microbiology (SYNMIKRO) and Department of Biology, Philipps-Universität Marburg, 35043 Marburg, Germany

[b] Max Planck Institute for Terrestrial Microbiology, 35043 Marburg, Germany

[#]For correspondence: anke.becker@synmikro.uni-marburg.de

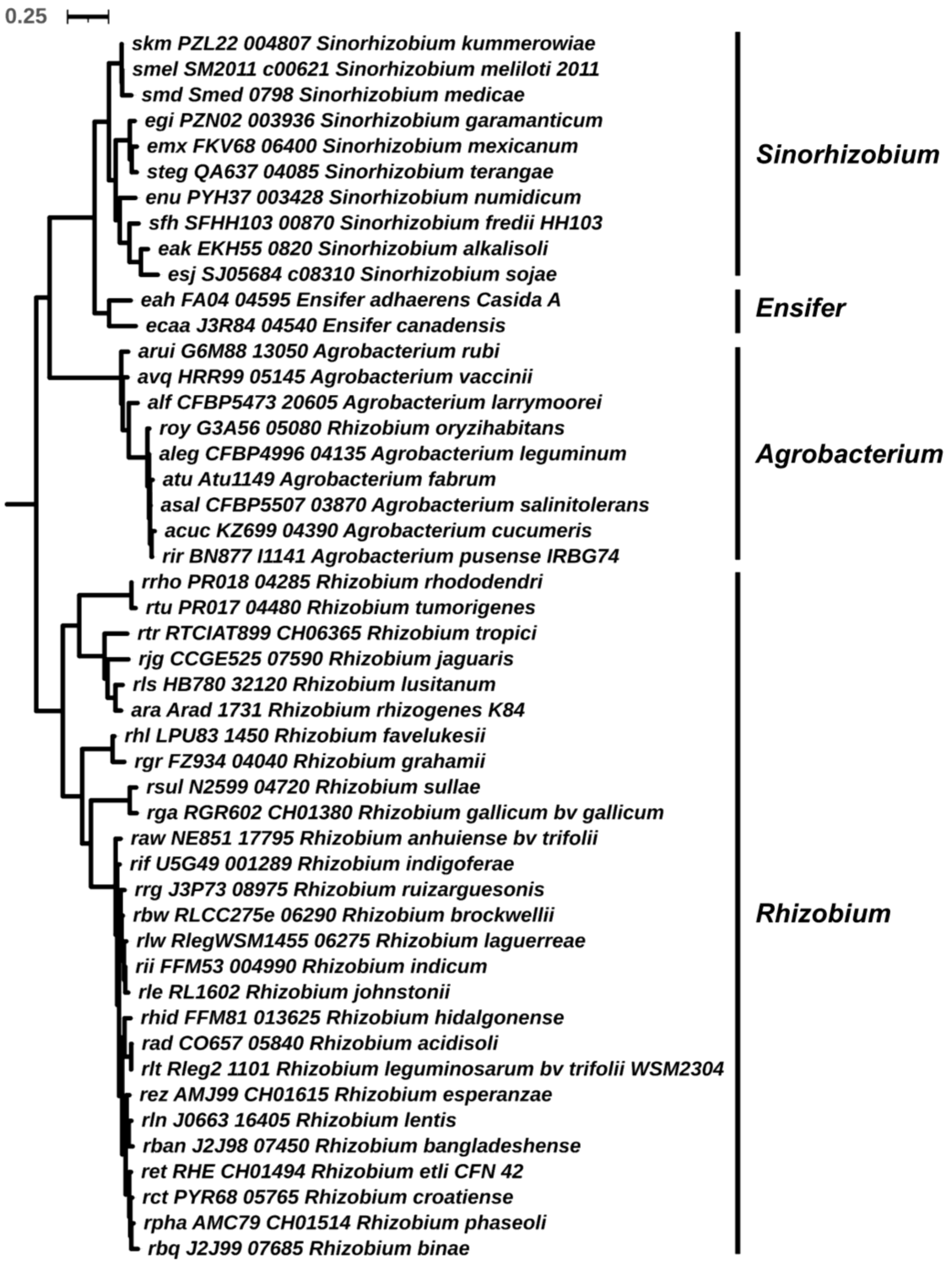

Figure S1. **Phylogenetic tree representing the relationship of CyaB orthologs from 48 species of four different genera of the *Rhizobiaceae*, compared to CyaB from *S. meliloti* Rm2011.** BLASTP within the KEGG genome database was executed using the BLOSUM62 matrix and sequences downloaded in FASTA format. The phylogenetic tree was generated via the NGPhylogeny online tool (https://ngphylogeny.fr/).

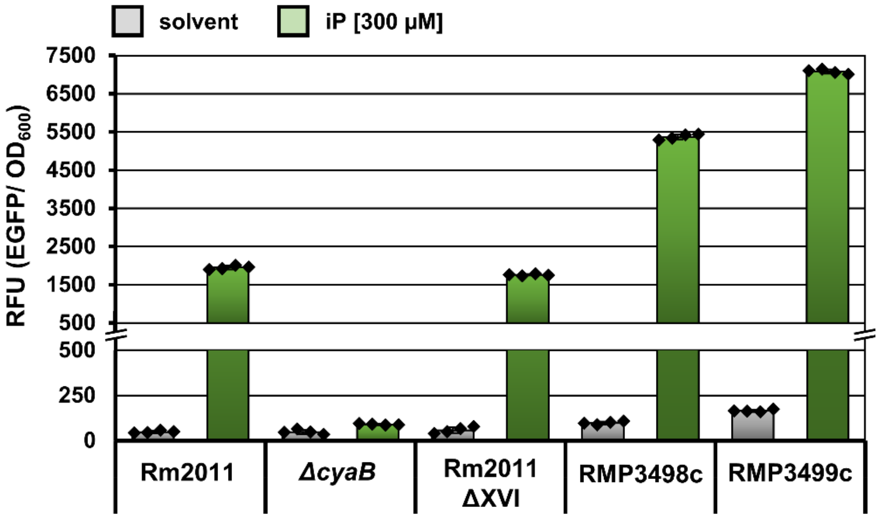

Figure S2. The CHASE domain containing diguanylate cyclase/ phosphodiesterase genes and genes located on pSymA are not controlling the iP-mediated cAMP signaling. EGFP reporter fluorescence of indicated *S. meliloti* strains, carrying the reporter plasmid pSRKKm-smc02178-EGFP. The strain RMP3498c is a pSymA-deficient derivative of the wild type-like strain RMP3499c. Rm2011 *Δ*XVI is lacking 16 cyclic di-GMP synthases including Smc03178 and Sma0137, containing CHASE and CHASE4 domains, respectively. The strains were grown for 24 h in Vincent minimal medium supplemented with either solvent or 300 µM iP. The error bars represent the standard deviation of four biological replicates and the diamonds show the single values.

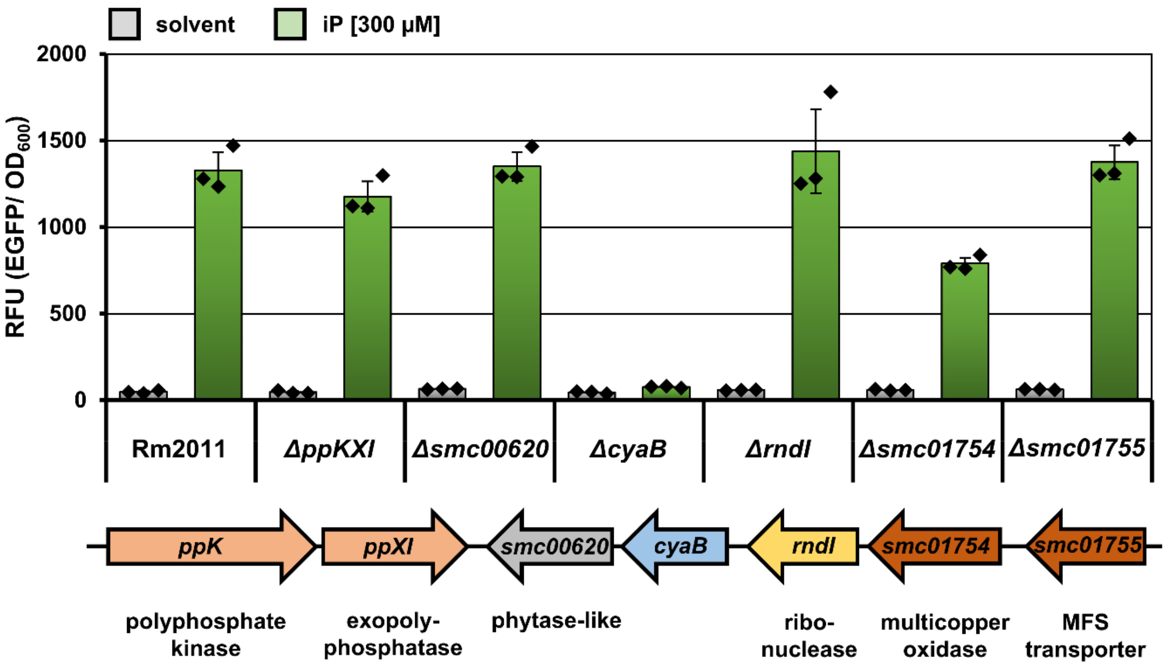

Figure S3. **The genes encoded in the chromosome in the vicinity of *cyaB* are not involved in the iP-mediated cAMP signaling.** a) Reporter fluorescence of the Rm2011 wild type, *ΔcyaB* and the strains with deletions in the indicated genes, located on the chromosome in the vicinity of *cyaB*. All strains carried the reporter plasmid pSRKKm-smc02178-EGFP. The strains were grown for 24 h in Vincent minimal medium supplemented with either solvent or 300 µM iP. The error bars represent the standard deviation of three biological replicates and the diamonds show the single values. The genes located on the chromosome in the vicinity of *cyaB* are shown underneath.

Table S1. Proteins identified as potential CyaB interaction partners in co-immunoprecipitation samples derived from cultures growth without iP. Shown are 20 top-ranked proteins, reliably detected in the CyaB-C3F co-immunoprecipitation samples, but not in the negative control samples.

Accession	Gene ID	Gene name	Description	Rank	Score Sequest HT	Coverage [%]	# Peptides	# PSMs	# AAs	MW [kDa]	calc. pI
NP_385296.1	SMc00621	CyaB	Adenylate cyclase 2 protein	1	106.94	41	10	33	363	39.9	8.16
NP_384986.1	SMc00972	Dxs	1-deoxy-D-xylulose-5-phosphate synthase	2	50.86	24	8	13	645	68.6	6.47
NP_384420.1	SMc00394	GuaA	GMP synthase	3	28.92	26	8	8	520	57.2	6.11
NP_386959.1	SMc03931	SoxA2	Sarcosine oxidase subunit alpha	4	19.63	7	4	5	997	107.5	6.92
NP_384204.3	SMc04154	PurU2	Formyltetrahydrofolate deformylase	5	15.91	14	2	4	296	32.9	7.02
NP_386976.1	SMc02978	SMc02978	Hypothetical protein	6	14.85	18	4	4	368	41.8	5.72
NP_385878.1	SMc00495	PurC	Phosphoribosylaminoimidazolesuccinocarboxamide synthase	7	13.11	21	4	4	254	29	6.01
NP_386966.1	SMc03938	PntB	NAD(P) transhydrogenase subunit beta	8	12.16	9	2	3	466	48.3	6.54
NP_386788.1	SMc00690	AccA	Acetyl-CoA carboxylase carboxyltransferase subunit alpha	9	10.81	15	3	3	317	34.7	6.46
NP_384375.1	SMc00349	LepA	GTP-binding protein LepA	10	9.5	7	3	3	608	67.1	5.82
NP_386281.1	SMc01867	MurC	UDP-N-acetylmuramate--L-alanine ligase	11	8.46	9	3	3	471	50.9	6.98
NP_385434.1	SMc01336	Rne	Ribonuclease E protein	12	7.8	4	3	3	924	102.2	5.15
NP_384139.1	SMc02761	TrxA	Thioredoxin	13	7.17	30	2	2	107	11.4	4.93
NP_385363.1	SMc01905	Lon	ATP-dependent protease LA protein	14	6.9	4	2	2	806	89.4	6.28
NP_387160.1	SMc02481	SucD	Succinyl-CoA synthetase subunit alpha	15	6.75	9	2	2	300	30.9	7.33
NP_386295.1	SMc01853	SMc01853	Hypothetical protein	16	6.53	11	2	2	235	26.3	6.04
NP_386238.1	SMc01449	SMc01449	Hypothetical protein	17	6.27	25	2	2	102	11.2	8.63
NP_385248.1	SMc00572	FabG	3-ketoacyl-ACP reductase	18	6.27	11	2	2	245	25.9	7.46
NP_386157.1	SMc04296	FtsZ2	Cell division protein FtsZ	19	6.13	8	2	2	346	36.1	4.87
NP_437785.1	SMb21418	SMb21418	NDP-hexose 3-C-methyltransferase	20	6.1	9	2	2	410	44.8	5.68

Table S2. Proteins identified as potential CyaB interaction partners in co-immunoprecipitation samples derived from cultures growth with 300 µM iP. Shown are 20 top-ranked proteins, reliably detected in the CyaB-C3F co-immunoprecipitation samples, but not in the negative control samples.

Accession	Gene ID	Protein name	Description	Rank	Average Score Sequest HT	Average coverage [%]	Average # Peptides	Average # PSMs	# AAs	MW [kDa]	calc. pI
NP_385296.1	SMc00621	CyaB	Adenylate cyclase 2 protein	1	283	52	17	85	363	39.9	8.16
NP_384986.1	SMc00972	Dxs	1-deoxy-D-xylulose-5-phosphate synthase	2	244	55	21	72	645	68.6	6.47
NP_387392.1	SMc04382	NdvB	Beta-(1,2)-glucan production associated transmembrane protein	3	168	24	42	56	2832	315.6	6.11
NP_384572.1	SMc02181	PutA	Bifunctional proline dehydrogenase/pyrroline-5-carboxylate dehydrogenase	4	128	34	24	38	1233	131.5	6.49
NP_386959.1	SMc03931	SoxA2	Sarcosine oxidase subunit alpha	5	127	35	23	38	997	107.5	6.92
NP_386733.1	SMc04458	SecA	Preprotein translocase subunit SecA	6	103	38	21	35	903	101.5	5.29
NP_437113.1	SMb20830	KpsF2	Arabinose-5-phosphate isomerase	7	97	38	11	27	337	35.1	6.1
NP_437626.1	SMb20961	ExoP	Protein tyrosine kinase MPA1 family protein	8	84	32	16	27	786	86.1	7.34
NP_385762.1	SMc00231	GlmS	Glucosamine--fructose-6-phosphate aminotransferase	9	79	39	15	24	608	65.8	6
NP_384136.1	SMc02764	AccD	Acetyl-CoA carboxylase subunit beta	10	78	59	12	26	304	33.5	8.62
NP_386788.1	SMc00690	AccA	Acetyl-CoA carboxylase carboxyltransferase subunit alpha	11	71	49	10	21	317	34.7	6.46
NP_386094.1	SMc04347	SMc04347	hypothetical protein SMc04347	12	62	**20**	10	20	683	73.9	6.55
NP_436456.1	SMa2245	SMa2245	Hypothetical protein	13	61	28	15	22	845	94.7	6.9
NP_385622.1	SMc02080	ValS	Valyl-tRNA synthetase	14	61	21	13	19	947	107	5.27
NP_386229.1	SMc01440	HflC	Hydrolase serine protease transmembrane protein	15	57	41	10	18	310	35.1	5.25
NP_385922.1	SMc00190	SMc00190	hypothetical protein SMc00190	16	57	**14**	14	18	2089	222.4	4.94
NP_384560.1	SMc01712	LldD2	L-lactate dehydrogenase (cytochrome) protein	17	54	35	11	17	403	44.9	8.35
NP_387162.1	SMc02479	Mdh	Malate dehydrogenase	18	54	45	11	18	320	33.6	6.04
NP_386307.1	SMc04454	SMc04454	ABC transporter ATP-binding protein	19	53	34	12	16	549	60.9	5.34
NP_437117.1	SMb20834	RkpZ1	Surface saccharide synthesis protein	20	52	42	12	17	432	48.9	8.81

Table S3: Strains and plasmids used in this study

Strain	Description	Reference
E. coli		
DH5α	F^- *endA1 supE44 thi-1I-recA1 gyrA96 relA1 deoRD(lacZYA-argF)*U169 Nxs Strs Rifs Thi- Lac- Ara+Gal+ Mtl- F- RecA+ Uvr+ Lon+	Grant *et al.* 1990
MT616	*E. coli* MT607 (*pro-82 thi-I hsdR17 supE44 recA56*) containing the pRK600 helper plasmid for triparental mating, Cm^r	Finan *et al.* 1986
BTH101	F^- *cya-99 araD139 galE15 galK16 rpsL1 (str^r) hsdR2 mcrA1 mcrB1*	Karimova *et al.* 2005
S. meliloti		
Rm2011	Wild type, Str^r	Casse *et al.* 1979
Δ*cyaA*	Rm2011 with markerless deletion of *cyaA*	This work
Δ*cyaB*	Rm2011 with markerless deletion of *cyaB*	This work
Δ*cyaC*	Rm2011 with markerless deletion of *cyaC*	This work
Δ*cyaD1D2K*	Rm2011 with markerless deletions of *cyaD1, cyaD2* and *cyaK*	This work
Δ*cyaE1*	Rm2011 with markerless deletion of *cyaE1*	This work
Δ*cyaE2*	Rm2011 with markerless deletion of *cyaE2*	This work
Δ*cyaF1-F7*	Rm2011 with markerless deletions of *cyaF1, cyaF2, cyaF3, cyaF4, cyaF5, cyaF6* and *cyaF7*	This work
Δ*cyaG1G2HMO*	Rm2011 with markerless deletions of *cyaG1, cyaG2, cyaH, cyaM* and *cyaO*	This work
Δ*cyaI1-4*	Rm2011 with markerless deletions of *cyaI1, cya2, cya3* and *cya4*	This work
Δ*cyaJ*	Rm2011 with markerless deletion of *cyaJ*	This work
Δ*cyaL*	Rm2011 with markerless deletion of *cyaL*	This work
Δ*cyaN*	Rm2011 with markerless deletion of *cyaN*	This work
Δ*cyaP*	Rm2011 with markerless deletion of *cyaP*	This work
cya^0	Rm2011 Δ*cyaABCD1D2E1E2F1-7G1G2HI1-4JKLMNOP*, markerless deletions of 28 AC/GC genes	Werel et al. 2023
cya^0*::cyaB*	Rm2011 cya^0, $cyaB_{Sm}$ reintegrated into the chromosome at the native *cyaB* locus	This work
Δ*cyaB::cyaB*	Rm2011 Δ*cyaB*, $cyaB_{Sm}$ reintegrated into the chromosome at the native *cyaB* locus	This work
Δ*cyaB::fa04_04595*	Rm2011 Δ*cyaB*, *fa04_04595* ($cyaB_{Ea}$) integrated into the chromosome at the native *cyaB* locus	This work
Δ*cyaB::rl1602*	Rm2011 Δ*cyaB*, *rl1602* ($cyaB_{Rj}$) integrated into the chromosome at the native *cyaB* locus	This work
Δ*cyaB::atu1149*	Rm2011 Δ*cyaB*, *atu1149* ($cyaB_{Af}$) integrated into the chromosome at the native *cyaB* locus	This work
Δ*ppKXI*	Rm2011 with markerless deletion of *ppKXI*	This work
Δ*smc00620*	Rm2011 with markerless deletion of *smc00620*	This work

Δ*rndI*	Rm2011 with markerless deletion of *rndI*	This work
Δ*smc01754*	Rm2011 with markerless deletion of *smc01754*	This work
Δ*smc01755*	Rm2011 with markerless deletion of *smc01755*	This work
Rm2011 ΔXVI	Rm2011 with markerless deletions of *pleD, smc04015, smb20523, smc01464, sma2301, smb20389, smb2044, smb20900, smc00038, sma1548, smc03178, sma0137, smc00992, smc03942, smc00887* and *smc00033*	Schäper *et al*. 2016
RMP3498	Rm2011 ΔpSymAB with pSymB reintroduced into the genome, pSymA-deficient, [1]	diCenzo *et al*. 2016
RMP3499	Rm2011 ΔpSymAB with pSymA and pSymB reintroduced into the genome, wild type-like, [1]	diCenzo et al. 2016
RMP3498c	RMP3498 with *clr* from Rm2011 integrated into the chromosome at the native *clr* locus	This work
RMP3499c	RMP3499 with *clr* from Rm2011 integrated into the chromosome at the native *clr* locus	This work
**Plasmids**		
pSRKKm-EGFP	pSRKKm, contains *eGFP* coding sequence, Km^r	Schäper *et al*. 2016
pSRKKm-smc02178-EGFP	pSRKKm-eGFP, contains $P_{smc02178}$ promoter sequence, Km^r	Krol *et al.* 2016
pSRKKm-3xFLAG	pSRKKm, contains 3x-FLAG coding sequence, Km^r	B. Schlachter
pSRKKm-cyaB-C3F	pSRKKm-3xFLAG, contains *cyaB* coding sequence, Km^r	This work
pUT18Spe	pUT18 with a SpeI restriction site upstream of T18 start codon, Amp^r	Schäper *et al.* 2018
pKNT25Spe	pKNT25 with a SpeI restriction site upstream of T25 start codon, Km^r	Krol *et al*. 2020
pUT18C-Zip	pUT18C carrying the leucine zipper of GCN4, Amp^r	Karimova *et al.* 1998
pKT25-Zip	pKT25 carrying the leucine zipper of GCN4, Km^r	Karimova *et al.* 1998
pUT18-cyaB*$_{Sm}$	pUT18Spe, contains *S. meliloti cyaB* coding sequence with amino acid substitutions D162H, G205A and D206H, Amp^r	This work
pKNT25-cyaB*$_{Sm}$	pKNT25Spe, contains *S. meliloti cyaB* coding sequence with amino acid substitutions D162H, G205A and D206H, Km^r	This work
pUT18-cyaB*$_{Ea}$	pUT18Spe, contains coding sequence of *E. adherens cyaB* homolog *fa04_04595* with amino acid substitutions D162H, G205A and D206H, Amp^r	This work
pKNT25-cyaB*$_{Ea}$	pKNT25Spe, contains coding sequence of *E. adherens cyaB* homolog *fa04_04595* with amino acid substitutions D162H, G205A and D206H, Km^r	This work
pUT18-cyaB*$_{Rj}$	pUT18Spe, contains coding sequence of *R.jonstonii cyaB* homolog *rl1602* with amino acid substitutions D164H, G207A and D208H, Amp^r	This work
pKNT25-cyaB*$_{Rj}$	pKNT25Spe, contains coding sequence of *R.jonstonii cyaB* homolog *rl1602* with amino acid substitutions D164H, G207A and D208H, Km^r	This work
pUT18-cyaB*$_{Af}$	pUT18Spe, contains coding sequence of *A. fabrum cyaB* homolog *atu1149* with amino acid substitutions D162H, G205A and D206H, Amp^r	This work

pKNT25-cyaB*$_{Af}$	pKNT25Spe, contains coding sequence of *A. fabrum cyaB* homolog *atu1149* with amino acid substitutions D162H, G205A and D206H, Km^r	This work
pUT18-dxs	pUT18Spe, contains *S. meliloti dxs* coding sequence, Amp^r	This work
pKNT25-dxs	pKNT25Spe, contains *S. meliloti dxs* coding sequence, Km^r	This work
pK18mobsacB	High copy number vector, contains *lacZ* and *sacB* coding sequence, mob site for conjugation, used for gene deletion/ insertion, Km^r	Schäfer *et al*. 1994
pK18mobsacB-clr	pK18mobsacB, contains *clr* coding sequence with additional flanking regions for homologous recombination into Rm2011 chromosome, Km^r	This work
pK18mobsacB-cyaB	pK18mobsacB, contains *cyaB* coding sequence with additional flanking regions for homologous recombination into Rm2011 chromosome at the native *cyaB* locus, Km^r	This work
pK18mobsacB-fa04_04595	pK18mobsacB, contains *fa04_04595* coding sequence with additional flanking regions for homologous recombination into Rm2011 chromosome at the native *cyaB* locus, Km^r	This work
pK18mobsacB-rl1602	pK18mobsacB, contains *rl1602* coding sequence with additional flanking regions for homologous recombination into Rm2011 chromosome at the native *cyaB* locus, Km^r	This work
pK18mobsacB-atu1149	pK18mobsacB, contains *atu1149* coding sequence with additional flanking regions for homologous recombination into Rm2011 chromosome at the native *cyaB* locus, Km^r	This work
pK18mobsacB-Δppxk1	pK18mobsacB, contains flanking regions of *ppx* and *ppk1* for gene deletion, Km^r	This work
pK18mobsacB-Δsmc00620	pK18mobsacB, contains flanking regions of *smc00620* for gene deletion, Km^r	This work
pK18mobsacB-ΔrndI	pK18mobsacB, contains flanking regions of *rndI* for gene deletion, Km^r	This work
pK18mobsacB-Δsmc01754	pK18mobsacB, contains flanking regions of *smc01754* for gene deletion, Km^r	This work
pK18mobsacB-Δsmc01755	pK18mobsacB, contains flanking regions of *smc01755* for gene deletion, Km^r	This work
pK18mobsacB ΔcyaA	pK18mobsacB, contains flanking regions of *cyaA* for gene deletion, Km^r	Werel *et al.* 2023
pK18mobsacB ΔcyaB	pK18mobsacB, contains flanking regions of *cyaB* for gene deletion, Km^r	Werel *et al.* 2023
pK18mobsacB ΔcyaC	pK18mobsacB, contains flanking regions of *cyaC* for gene deletion, Km^r	Werel *et al.* 2023
pK18mobsacB ΔcyaD1	pK18mobsacB, contains flanking regions of *cyaD1* for gene deletion, Km^r	Werel *et al.* 2023
pK18mobsacB ΔcyaD2	pK18mobsacB, contains flanking regions of *cyaD2* for gene deletion, Km^r	Werel *et al.* 2023
pK18mobSacB ΔcyaE1	pK18mobsacB, contains flanking regions of *cyaE1* for gene deletion, Km^r	Werel *et al.* 2023
pK18mobSacB ΔcyaE2	pK18mobsacB, contains flanking regions of *cyaE2* for gene deletion, Km^r	Werel *et al.* 2023
pK18mobsacB ΔcyaF1	pK18mobsacB, contains flanking regions of *cyaF1* for gene deletion, Km^r	Werel *et al.* 2023
pK18mobsacB ΔcyaF2	pK18mobsacB, contains flanking regions of *cyaF2* for gene deletion, Km^r	Werel *et al.* 2023
pK18mobsacB ΔcyaF3	pK18mobsacB, contains flanking regions of *cyaF3* for gene deletion, Km^r	Werel *et al.* 2023

pK18mobsacB ΔcyaF4	pK18mobsacB, contains flanking regions of *cyaF4* for gene deletion, Km^r	Werel *et al.* 2023
pK18mobsacB ΔcyaF5	pK18mobsacB, contains flanking regions of *cyaF5* for gene deletion, Km^r	Werel *et al.* 2023
pK18mobsacB ΔcyaF6	pK18mobsacB, contains flanking regions of *cyaF6* for gene deletion, Km^r	Werel *et al.* 2023
pK18mobsacB ΔcyaF7	pK18mobsacB, contains flanking regions of *cyaF7* for gene deletion, Km^r	Werel *et al.* 2023
pK18mobsacB ΔcyaG1	pK18mobsacB, contains flanking regions of *cyaG1* for gene deletion, Km^r	Werel *et al.* 2023
pK18mobsacB ΔcyaG2	pK18mobsacB, contains flanking regions of *cyaG2* for gene deletion, Km^r	Werel *et al.* 2023
pK18mobsacB ΔcyaH	pK18mobsacB, contains flanking regions of *cyaH* for gene deletion, Km^r	Werel *et al.* 2023
pK18mobsacB ΔcyaI1	pK18mobsacB, contains flanking regions of *cyaI1* for gene deletion, Km^r	Werel *et al.* 2023
pK18mobsacB ΔcyaI2	pK18mobsacB, contains flanking regions of *cyaI2* for gene deletion, Km^r	Werel *et al.* 2023
pK18mobsacB ΔcyaI3	pK18mobsacB, contains flanking regions of *cyaI3* for gene deletion, Km^r	Werel *et al.* 2023
pK18mobsacB ΔcyaI4	pK18mobsacB, contains flanking regions of *cyaI4* for gene deletion, Km^r	Werel *et al.* 2023
pK18mobsacB ΔcyaJ	pK18mobsacB, contains flanking regions of *cyaJ* for gene deletion, Km^r	Werel *et al.* 2023
pK18mobsacB ΔcyaK	pK18mobsacB, contains flanking regions of *cyaK* for gene deletion, Km^r	Werel *et al.* 2023
pK18mobsacB ΔcyaL	pK18mobsacB, contains flanking regions of *cyaL* for gene deletion, Km^r	Werel *et al.* 2023
pK18mobsacB ΔcyaM	pK18mobsacB, contains flanking regions of *cyaM* for gene deletion, Km^r	Werel *et al.* 2023
pK18mobsacB ΔcyaN	pK18mobsacB, contains flanking regions of *cyaN* for gene deletion, Km^r	Werel *et al.* 2023
pK18mobsacB ΔcyaO	pK18mobsacB, contains flanking regions of *cyaO* for gene deletion, Km^r	Werel *et al.* 2023
pK18mobsacB ΔcyaP	pK18mobsacB, contains flanking regions of *cyaP* for gene deletion, Km^r	Werel *et al.* 2023

Table S4. Oligonucleotides used in this study

Name	5'-3' Sequence	Purpose	Target Vector
cyaB Prom NdeI fw	CATCCATATGAGCTCTTCGGCATGACGG	Co-IP constructs	pSRKKm
cyaB noStop XbaI re	CATCTCTAGACAGCGAGGTGAAAGCCTCG		
cyaB XbaI fw	CATCTCTAGAATGCGCTGGAGATTTTCCAC	BTH constructs, overlap PCR to introduce point mutations into *cyaB* coding sequence	pUT18Spe/ pKNT25Spe
cyaB noSTOP frameshift KpnI re	CATCGGTACCCGCAGCGAGGTGAAAGCCTCG		
CyaB ACYC D162H re	CGATCCGGCGAGATGGATGAAAA		
CyaB ACYC D162H fw	TTTTCATCCATCTCGCCGGATCG		
CyaB ACYC G205A D206H re	ACGGCAGCGTGGGCGACATAGTCGTCGAT		
CyaB ACYC G205A D206H fw	GACTATGTCGCCCACGCTGCCGTGATCA		
FA04_04595 XbaI fw	CATCTCTAGAATGCGTTGGCGCTTTTCGACTTTCGAAG		
FA04_04595 noSTOP Frameshift KpnI re	CATCGGTACCCGCTCGGCCGCGGTCGATGC		
FA04_04595 D162H fw	CCTCTTCATCCACCTGGCCGGCT		
FA04_04595 D162H re	AGCCGGCCAGGTGGATGAAGAGG		
FA04_04595 G205A D206H fw	CGACTATATCGCTCACGCCGCCGTCA		
FA04_04595 G205A D206H re	TGACGGCGGCGTGAGCGATATAGTCG		
RL1602 XbaI fw	CATCTCTAGAATGCGGAAATATCTCCGACACAGAATTGGA		
RL1602 noSTOP frameshift KpnI re	CATCGGTACCCGGCCGTGCAGTATGACCGCCTG		
RL1602 D164H fw	CCTGTTCATCCACCTCGTCGATT		
RL1602 D164H re	AATCGACGAGGTGGATGAACAGG		
RL1602 G207A D208H fw	CGACTATGTCGCCCATGCGGCGATCA		
RL1602 G207A D208H re	TGATCGCCGCATGGGCGACATAGTCG		
Atu1149 XbaI fw	CATCTCTAGAATGAGAAAATTGCTGCCCATTCTGGATT		
Atu1149 noSTOP Frameshift KpnI re	CATCGGTACCCGTGCGGCAGGGGCCTGATAT		
Atu1149 D162H fw	CATCTTCATCCATCTCGTCGGCT		
Atu1149 D162H re	AGCCGACGAGATGGATGAAGATG		
Atu1149 G205A D206H fw	CGATTACATTGCCCATGCCGCCATCAT		
Atu1149 G205A D206H re	ATGATGGCGGCATGGGCAATGTAATCG		
Dxs XbaI fw	CATCTCTAGAGTGACACAACTGCCAACCAACCCGATGCCGG		
Dxs noSTOP frameshift KpnI re	CATCGGTACCCGGCCGGCGGCGCCGAGGCCGACG		

cyaB 500 up XbaI fw	CATCTCTAGACGAGCTGCCGCGCCTGCCGC	Chromosomal integration constructs	pK18mobsacB
cyaB 500 down HindIII re	CATCAAGCTTGGTCTTCAACGCCTCGACCCTGCCGG		
cyaB 500 up HindIII fw	CATCAAGCTTCGAGCTGCCGCGCCTGCCGC		
PcyaB XbaI re	CATCTCTAGAGTGACAGCCTCGAAATCGAGAATT		
FA04_04595 XbaI fw	CATCTCTAGAATGCGTTGGCGCTTTTCGACTTTCGAAG		
FA04_04595 Acc65I re	CATCGGTACCTCACTCGGCCGCGGTCGAT		
RL1602 XbaI fw	CATCTCTAGAATGCGGGAAATATCTCCGACACAGAATTGGA		
RL1602 Acc65I re	CATCGGTACCTCAGCCGTGCAGTATGACCGCC		
cya Atu1149 XbaI fw	CATCTCTAGAATGAGAAAATTGCTGCCCATTCTG		
cya Atu1149 Acc65I re	CATCGGTACCTTATGCGGCAGGGGCCTG		
cyaB down Acc65I fw	CATCGGTACCGACAAACATCAAACAGTTGTCATCG		
cyaB 500 down BamHI re	CATCGGATCCTCTTCAACGCCTCGACCC		
clr 235up EcoRI fw	CATCGAATTCGCGCGAGGCTCCTGTTGG		
clr 61down BamHI re	CATCGGATCCCCTCGGCCTTACGCTCAACC		
ppxI ppk HR1 HindIII fw	CATCAAGCTTCGTCGACAAGGCCTGCGC	Gene deletion constructs	
ppxI ppk HR1 XbaI re	CATCTCTAGATTGCTCTTTCGAACGTTCGCGC		
ppxI ppk HR2 XbaI fw	CATCTCTAGAGCCTGTAAAGCCCCATTACTCCGAAC		
ppxI ppk HR2 BamHI re	CATCGGATCCCGCCGCGGAAAAGCCGGTCC		
smc00620 HR1 HindIII fw	CATCAAGCTTAGCCTGCATCCGCTGCGTG		
smc00620 HR1 XbaI re	CATCTCTAGAAACTTCACCTCGGGTTGGAGTGCA		
smc00620 HR2 XbaI fw	CATCTCTAGAACCCACTCCTCCTGCCCTCATC		
smc00620 HR2 BamHI re	CATCGGATCCGCACTCGGCCTTCACGGG		
rnd1 HR1 HindIII fw	CATCAAGCTTTTTCCACGGCCAAGCGTATTCCG		
rnd1 HR1 XbaI re	CATCTCTAGACGGGCTTTAATCACTGTGGCAAAAATGC		
rnd1 HR2 XbaI fw	CATCTCTAGAAAACCGCCGCTCGACGACA		
rnd1 HR2 BamHI re	CATCGGATCCCGCCGGCGACGTGCAAAA		
smc01754 HR1 HindIII fw	CATCAAGCTTGCGGGCGGCCAGACGCGG		
smc01754 HR1 XbaI re	CATCTCTAGACGGTATCTTTGCGTGTCTATTTCCGG		
smc01754 HR2 XbaI fw	CATCTCTAGACAATTTCGATTGTGACAGGTGCCG		
smc01754 HR2 BamHI re	CATCGGATCCGGTCTGCGGCTCCAATCGGTGAA		
smc01755 HR1 HindIII fw	CATCAAGCTTGCACGGTCTCGGCGGTCT		
smc01755 HR1 XbaI re	CATCTCTAGAGGAACAGAAGGCATCCGATTGCGG		
smc01755 HR2 XbaI fw	CATCTCTAGATCCGATAGGGCTGCAGCTAGAGC		
smc01755 HR2 BamHI re	CATCGGATCCCCGCGAAGGGCTCGCCCT		

References

Casse F, Boucher C, Julliot JS, Michel M, Denarie J. 1979. Identification and characterization of large plasmids in *Rhizobium meliloti* using agarose-gel electorphoresis. J Gen Microbiol 113, 229-242. doi: https://doi.org/10.1099/00221287-113-2-229

diCenzo GC, Zamani M, Milunovic B, Finan TM. 2016. Genomic resources for identification of the minimal N2 -fixing symbiotic genome. Environ Microbiol 18(8):2534-47. doi: 10.1111/1462-2920.13221.

Grant SG, Jessee J, Bloom FR, Hanahan D. 1990. Differential plasmid rescue from transgenic mouse DNAs into *Escherichia coli* methylation-restriction mutants. Proc Natl Acad Sci U S A 87(12):4645-9. doi: 10.1073/pnas.87.12.4645.

Finan TM, Kunkel B, De Vos GF, Signer ER. 1986. Second symbiotic megaplasmid in *Rhizobium meliloti* carrying exopolysaccharide and thiamine synthesis genes. J Bacteriol 167(1):66-72. doi: 10.1128/jb.167.1.66-72.1986.

Karimova G, Dautin N, Ladant D. 2005. Interaction network among *Escherichia coli* membrane proteins involved in cell division as revealed by bacterial two-hybrid analysis. J Bacteriol 187(7):2233-43. doi: 10.1128/JB.187.7.2233-2243.2005.

Karimova G, Pidoux J, Ullmann A, Ladant D. 1998. A bacterial two-hybrid system based on a reconstituted signal transduction pathway. Proc Natl Acad Sci U S A 95(10):5752-6. doi: 10.1073/pnas.95.10.5752.

Khan SR, Gaines J, Roop 2nd RM, Farrand SK. 2008. Broad-host-range expression vectors with tightly regulated promoters and their use to examine the influence of TraR and TraM expression on Ti plasmid quorum sensing. Appl Environ Microbiol 74, 5053-5062. doi: 10.1128/AEM.01098-08.

Krol E, Klaner C, Gnau P, Kaever V, Essen LO, Becker A. 2016. Cyclic mononucleotide- and Clr-dependent gene regulation in *Sinorhizobium meliloti*. Microbiology 162, 1840–1856. doi: 10.1099/mic.0.000356.

Werel L, Farmani N, Krol E, Serrania J, Essen LO, Becker A. 2023. Structural Basis of Dual Specificity of *Sinorhizobium meliloti* Clr, a cAMP and cGMP Receptor Protein. mBio 14(2):e0302822. doi: 10.1128/mbio.03028-22.

Schäper S, Krol E, Skotnicka D, Kaever V, Hilker R, Søgaard-Andersen L, Becker A. 2016. Cyclic di-GMP regulates multiple cellular functions in the symbiotic alphaproteobacterium *Sinorhizobium meliloti*. J Bacteriol 198, 521-535. doi: 10.1128/JB.00795-15.

Schäfer A, Tauch A, Jäger W, Kalinowski J, Thierbach G, Pühler A. 1994. Small mobilizable multi-purpose cloning vectors derived from the *Escherichia coli* plasmids pK18 and pK19: selection of defined deletions in the chromosome of *Corynebacterium glutamicum.* Gene 145(1):69-73. doi: 10.1016/0378-1119(94)90324-7.

Schäper S, Yau HCL, Krol E, Skotnicka D, Heimerl T, Gray J, Kaever V, Søgaard-Andersen L, Vollmer W, Becker A. 2018. Seven-transmembrane receptor protein RgsP and cell wall-binding protein RgsM promote unipolar growth in Rhizobiales. PLoS Genet. 14(8):e1007594. doi: 10.1371/journal.pgen.1007594.
